# Autocrine Regulation of Macrophage Activation via Exocytosis of ATP and Activation of P2Y11 Receptor

**DOI:** 10.1371/journal.pone.0059778

**Published:** 2013-04-05

**Authors:** Hayato Sakaki, Mitsutoshi Tsukimoto, Hitoshi Harada, Yoshinori Moriyama, Shuji Kojima

**Affiliations:** 1 Department of Radiation Biosciences, Faculty of Pharmaceutical Sciences, Tokyo University of Science, Noda-shi Chiba, Japan; 2 Faculty of Pharmaceutical Sciences, Suzuka University of Medical Science, Suzuka-shi, Mie, Japan; 3 Department of Membrane Biochemistry, Okayama University Graduate School of Medicine, Dentistry, and Pharmaceutical Sciences, Okayama, Japan; National Institute of Infectious Diseases, Japan

## Abstract

It is important to understand the mechanisms that regulate macrophage activation to establish novel therapies for inflammatory diseases, such as sepsis; a systemic inflammatory response syndrome generally caused by bacterial lipopolysaccharide (LPS). In this study, we investigated the involvement of extracellular ATP-mediated autocrine signaling in LPS-induced macrophage activation. We show here that ATP release via exocytosis, followed by activation of P2Y11 receptor, is a major pathway of the macrophage activation, leading to release of cytokines. Treatment of human monocyte THP-1 cells with LPS induced rapid ATP release from cells, and this release was blocked by knockdown of SLC17A9 (vesicular nucleotide transporter, VNUT), which is responsible for exocytosis of ATP. ATP-enriched vesicles were found in cytosol of THP-1 cells. These data suggest the involvement of vesicular exocytosis in the release of ATP. Knockdown of SLC17A9, the P2Y11 antagonist NF157 or knockdown of P2Y11 receptor significantly suppressed both M1-type polarization and IL-6 production in THP-1 cells, indicating an important role of activation of P2Y11 receptor by released ATP in macrophage activation. Next, the effect of NF157 on LPS-induced immune activation was examined *in vivo*. Administration of LPS to mice caused increase of serum IL-1ß, IL-6, IL-12 and TNF-alpha levels at 3–24 h after the administration. Pre-treatment of LPS-treated mice with NF157 suppressed both elevation of proinflammatory cytokines in serum and M1 polarization of peritoneal/spleen macrophages. Moreover, post-treatment with NF157 at 30 min after administration of LPS also suppressed the elevation of serum cytokines levels. We conclude that vesicular exocytosis of ATP and autocrine, positive feedback through P2Y11 receptors is required for the effective activation of macrophages. Consequently, P2Y11 receptor antagonists may be drug candidates for treatment of inflammatory diseases such as sepsis.

## Introduction

Sepsis is a systemic inflammatory response syndrome induced by microbial infection. It is generally caused by Gram-negative bacterial endotoxins or lipopolysaccharide (LPS), and is characterized by severe shock and multiple organ failure [1], [2]. Although the pathogenesis of sepsis-induced lethal shock and multiple organ injury leading to death is incompletely understood, current theory holds that a hyperactive and out-of-balance network of endogenous proinflammatory cytokines contributes to mortality and morbidity in sepsis [3], [4]. Indeed, dysregulation in cytokine networking is thought to play a fundamental role in the outcome of sepsis. The current therapeutic strategies for human septic shock are designed to neutralize one or more of the inflammatory mediators, yet effective treatment modalities remain elusive [3], [5]. In fact, the incidence of sepsis is currently estimated to be ∼750,000 annually in the United States alone, with a mortality rate of about 30%, and its incidence is continuing to increase [6], [7].

Blood monocytes and macrophages play a central role in complex innate immune responses to infections, which influence the development of Th1/Th2 adaptive immunity [8]. Macrophages are highly versatile phagocytic cells whose diverse effector functions can be selectively reprogrammed by an array of environmental signals [9], [10], [11]. The two major macrophage phenotypes are the classic pro-inflammatory M1 and the alternative anti-inflammatory M2 phenotypes [12], [13]. The M1 phenotype is induced in response to microbial products such as LPS or proinflammatory cytokines, including IFN-gamma and TNF-alpha. M1 macrophages up-regulate proinflammatory cytokines, including TNF-alpha, interleukin (IL)-6, and IL-12, and are associated with increased production of reactive oxygen species (ROS) and nitrogen intermediates [14]. It has been demonstrated that M1 phenotype induction is related to the severity of sepsis [15]. In patients with severe sepsis, high concentrations of circulating M1-type cytokines are strongly correlated with mortality [16]. Macrophages from these patients produce high levels of type 1 cytokines and chemokines that activate the endothelium and contribute to cardiac failure, loss of general organ perfusion, and death [17]. To establish novel therapies for inflammatory diseases including sepsis, it is important to understand the mechanisms that regulate macrophage activation. Although the mechanism of macrophage activation has been well investigated, an autocrine signaling in the activation process is not established.

Recently, extracellular nucleotides, such as ATP or UTP, have been identified as damage-associated molecular pattern molecules (DAMPs), which activate innate immune systems. Extracellular nucleotides activate P2 receptors, which are classified into two subfamilies, ionotropic P2X1-7 and G-protein coupled P2Y1-14 receptors [18]. Activation of P2 receptors is important for immune functions. For example, ATP-induced activation of P2X7 receptor plays a critical role in release of IL-1ß from LPS-primed macrophages [19]. ATP is released from macrophages stimulated with various pathogen-associated molecular patterns (PAMPs) and extracellular ATP is one of the most pleiotropic inflammasome activators [20], [21]. Although it is well known that large amounts of nucleotides are released from dead cells, living cells also release nucleotides via non-lytic mechanisms, such as maxianion channels, anion transporter, hemichannels, or exocytosis, in response to various stimuli [22]. Recently, we have shown that vesicular exocytosis of ATP and activation of P2X7 and P2Y6 receptors play an important role in activation of T cells [23]. However, although ATP release from macrophages has been reported [24], it has not yet been established whether this occurs via exocytosis. In this connection, SLC17A9 has been identified as a vesicular nucleotide transporter (VNUT), which plays a key role in vesicular storage of ATP [25]. It mediates the active accumulation of nucleotides, driven by an electrochemical gradient of protons across the membrane, generated by vacuolar proton-ATPase. SLC17A9 is responsible for exocytosis of ATP, in the form of ATP-containing vesicles, and is expressed in PC12 cells, type II taste cells, biliary epithelial cells, T lymphocytes, and lung cancer cells [25], [26], [27], [28], [29]. However, expression of SLC17A9 and vesicular exocytosis of ATP have not been reported in macrophages.

As we are interested in finding new therapeutic targets for treatment of inflammatory diseases such as sepsis, in this study we investigated the involvement of extracellular nucleotides-mediated autocrine signaling via exocytosis and activation of P2 receptors in macrophage activation. Our findings indicate that LPS-induced activation of macrophages is mediated, at least in part, by ATP release via exocytosis and activation of P2Y11 receptor, and this pathway is involved in the increased production of cytokines by activated macrophages. Therefore, we propose that P2Y11 antagonists may be candidate anti-inflammatory drugs for the treatment of sepsis or other inflammatory diseases.

## Materials and Methods

### Reagents and Antibody

RPMI1640 medium was purchased from Wako Pure Chemical. FBS was purchased from Biowest. Bafilomycin A, brefeldin A (BFA), carbenoxolone (CBX), clopidgrel, 18-glycyrrhetinic acid (18GA), flufenamic acid (FFA), glibenclamide, GdCl_3_, apyrase, PPADS, suramin, and LPS were purchased from Sigma-Aldrich. MRS2179, MRS2211, MRS2578, NF157, KH7 and A438079 were from Tocris Bioscience. NF449 was from Abcam. IFN-gamma was purchased from Roche Applied Science. Rabbit polyclonal anti-SLC17A9 antibody was generated by Prof. Moriyama (University of Okayama).

### Cell Culture

THP-1 human acute monocytic leukemia cells (ATCC) or mouse macrophage RAW264.7 cells (ATCC) were grown in RPMI1640 medium, supplemented with 10% fetal bovine serum, penicillin (100 units/mL) and streptomycin (100 µg/mL) in a humidified atmosphere of 5% CO_2_ in air at 37°C. THP-1 cells were cultured in RPMI 1640 media with 1 µg/ml LPS from *Escherichia coli* serotype 055:B5 (Sigma-Aldrich) and 1000 U/ml mouse IFN-gamma (R&D Systems) to generate M1 macrophages.

### Determination of Extracellular ATP Concentration

THP-1 cells were washed and resuspended in RPMI1640-based buffer, containing 102 mM NaCl, 5 mM KCl, 0.4 mM CaCl_2_, 0.4 mM MgSO_4_, 23.8 mM NaHCO_3_, 5.6 mM Na_2_HPO_4_, 11.1 mM glucose and 10 mM HEPES-NaOH (pH 7.4), then 40 µL aliquots of THP-1 cells (1.0×10^6^ cells/mL) were stimulated with LPS (10 µg/mL) and the extracellular ATP concentration was measured using ENLITEN® rLuciferase/Luciferin Reagent (Promega). At the indicated time points, each sample was centrifuged at 800×g for 1 min and 10 µL of the supernatant was collected for ATP determination. The luciferin-luciferase reagent (100 µL) was injected into the supernatant and chemiluminescence was measured with a WALLAC ARVO SX multilabel counter (PerkinElmer). ATP concentration in each sample was determined by comparing the luminescence of samples with those of standards in the range of 10^−6^ to 10^−10^ M.

### Quantification of Lactate Dehydrogenase Release

Release of lactate dehydrogenase (LDH) into cell culture supernatant was quantified with a Cytotoxicity Detection Kit (Roche Applied Science), according to the supplied instructions. THP-1 cells (1.0×10^6^ cells/mL) were incubated in a 96-well plate at 37°C for 30 min with LPS. At the end of incubation, supernatants were collected and the LDH content was measured. LDH release is expressed as a percentage of the total content determined by lysing an equal amount of cells with 1% Triton X-100.

### Fluorescence Imaging

For staining of intracellular ATP, LPS- and IFN-gamma-treated THP-1 cells were incubated for 1 h with 50 µM 2′−/3′-O-(N′-methylanthraniloyl)-ATP (MANT-ATP) and 5 µM quinacrine dihydrochloride in RPMI1640-based buffer at 37°C. Stained cells were analyzed using a confocal laser scanning microscope (TCS SP2; Leica) equipped with a HCX PLApo 63×1.32 NA oil objective lens. Leica confocal software (TCS SP2, version 2.6.1) was used for image acquisition and processing. Fluorescence of MANT-ATP was detected at 430–480 nm with excitation at 364 nm, and fluorescence of quinacrine dihydrochloride was detected at 510–530 nm with excitation at 488 nm.

### RT-PCR Analysis

Total RNA was isolated from THP-1 cells using a Fast Pure RNA kit (Takara Bio). The first-strand cDNA was synthesized from total RNA with PrimeScript Reverse Transcriptase (Takata Bio). Specific primers were designed with PrimerQuest^SM^ (Integrated DNA Technologies, Inc.) and synthesized by Sigma Genosys (Sigma-Aldrich). The sequences of specific primers for human SLC17A9 were 5′-ACA CAC GAG CAG AGA GGA ACA CAA-3′ (sense) and 5′-TTT CTG GCT GTT GTC TGA CTG GGA-3′ (antisense). GAPDH mRNA was determined as a positive control. PCR was carried out by incubating each cDNA sample with the primers (0.5 µM each), PrimeSTAR® HS DNA Polymerase (0.625 U: Takara Bio), and deoxynucleotide mix (0.2 mM each: Takara Bio). Amplification was carried out for 35 cycles (94°C for 30 sec, annealing at 60°C for 30 sec). The products were then subjected to 2% agarose gel electrophoresis. Bands were stained with ethidium bromide (Sigma-Aldrich) and photographed.

### Short Hairpin RNA Plasmid (shRNA) and siRNA Transfection for Knock-down

Transfection of THP-1 cells was performed using the SureSilencing™ shRNA plasmid Kit for human SLC17A9 (SABiosciences) or TriFECTa Kit for human P2Y11 receptor (Integrated DNA Technologies) and the Nucleofector System (Amaxa GmbH). Two different shRNA plasmids targeting SLC17A9, the negative control shRNA plasmid or the siRNA duplex oligonucleotides (10 nM) for reduction of human P2Y11 receptor were transfected by electroporation using the Amaxa system (Lonza) with Nucleofector solution V and Nucleofector program V-01. Seventy-two hours after transfection, reduction of expression of SLC17A9 and P2Y11 receptor was confirmed.

### Real-time RT-PCR

Total RNA was extracted from THP-1 cells and first-strand cDNA was synthesized as described above. The cDNA was used as the template for real-time PCR analysis: reactions were performed in a Stratagene Mx3000P® QPCR system (Agilent Technologies). Specific primers were designed with PrimerQuest^SM^ and synthesized by Sigma Genosys. The sequences of specific primers for human SLC17A9 were 5′-AGT CTG TGG TCT TTG CAT CAG CCT-3′ (sense) and 5′-TGT TGG CCA CAC CAA ACA GAA AGC-3′ (antisense). The sequences of specific primers for human P2Y11 were 5′-CGT GAG CTG AGC CAA TGA TGTG-3′ (sense) and 5′-GGG TGG GAA AGG CGA CTGC-3′ (antisense). RT^2^-qPCR primer assay for human IL-6 was purchased from Qiagen. GAPDH mRNA was determined as a positive control. Each sample was assayed in a 20 µl amplification reaction, containing cDNA, primer mixture (5 µM each of sense and antisense primers) and 2x KAPA SYBR® FAST qPCR Master Mix (Kapa Biosystems). The amplification program included 40 cycles of two steps, each comprising heating to 95°C and to 60°C, respectively. Fluorescent products were detected at the last step of each cycle. The obtained values were within the linear range of the standard curve and were normalized to yield the same amount of GAPDH mRNA expression.

### Western Blot Analysis

Total cellular membrane protein was lysed in 30 µL of PBS containing 10 mM HEPES-NaOH, pH 7.4, 1% Triton X100, 5 mM EDTA, 30 mM sodium pyrophosphate, 50 mM sodium fluoride, 1 mM sodium orthovanadate, 1.04 mM 4-(2-aminoethyl)benzenesulfonyl fluoride, 0.8 µM aprotinin, 21 µM leupeptin, 36 µM bestatin, 15 µM pepstatin A and 14 µM E-64. The protein content in each sample was determined using the Bio-Rad Protein Assay kit (Bio-Rad Laboratories). Equal amounts of protein lysate were dissolved in 2×sample buffer (50% glycerin, 2% SDS, 125 mM Tris, 10 mM DTT) and boiled for 10 min. Aliquots of samples containing 1 µg of protein were analyzed by means of 10% SDS-PAGE and bands were transferred onto a PVDF membrane. The blots were blocked overnight in TBST with 5% skim milk at 4°C, then incubated with rabbit polyclonal anti-SLC17A9 antibody (1∶5000) overnight at 4°C or with rabbit anti-rat P2X_7_ receptor antibody (1∶200) (Cell Signaling Technology, Inc.) for 90 min at room temperature as a loading control. After having been washed with TBST, blots were incubated with goat HRP-conjugated anti-rabbit IgG antibody (Cell Signaling Technology, Inc.) for 90 min at room temperature. The blots were again washed with TBST, and specific proteins were visualized by using ECL Western blotting detection reagents (GE Healthcare) according to the manufacturer’s instructions.

### Determination of Cytokines

THP-1 cells were stimulated with LPS (10 µg/mL). After incubation for 24 h, the culture supernatant was harvested for determination of IL-6. The concentration of IL-6 was measured by ELISA, as described below. A 96-well plate was coated with purified anti-human IL-6 mAb (1∶250) (eBioscience) and incubated overnight at 4°C, then the wells were washed with PBS containing 0.05% Tween-20, and nonspecific binding was blocked with PBS containing 1% BSA for 1 h at room temperature. The plate was washed, and the culture supernatant was added for 2 h at room temperature. The plate was washed again, and biotin-conjugated anti-human IL-6 mAb (1∶1000) (eBioscience) was added for 1 h at room temperature. The plate was further washed, and avidin-horseradish peroxidase (Sigma-Aldrich) was added. The plate was incubated for 30 min at room temperature, then washed, and 3,3′,5,5′-tetramethylbenzidine was added for a few minutes. The reaction was stopped by adding 5 N H_2_SO_4_. The absorbance at 450 nm was measured with an ImmunoReader NJ-2000 (Nihon InterMed). A standard curve was established with recombinant human IL-6 (15–1000 pg/mL), and the concentration of IL-6 was estimated from the standard curve.

### Measurement of cAMP Level

The cAMP level was quantified with a DetectX High Sensitive Direct Cyclic AMP Chemiluminescent Immunoassay Kit (Arbor Assays), according to the supplied instructions. THP-1 cells or RAW264.7 cells (1.0×10^6^ cells) were incubated in a 24 -well plate at 37°C for 10–30 min with ATP or LPS. At the end of incubation, cells were lyzed and the cAMP content was measured.

### Preparation of Peritoneal Macrophages and in vitro Stimulation

Peritoneal cells were isolated by washing the peritoneal cavity with ice-cold PBS. The cells were cultured for 1 h and non-adherent cells were washed out with PBS. Adherent cells (peritoneal macrophages: 5×10^6^) were cultured with LPS (1 µg/ml) in RPMI 1640 medium with 10% FBS for 24 h. Concentrations of IL-6 and TNF-alpha in the culture supernatants were measured by ELISA.

### Flow Cytometric Analysis of Macrophage Polarization

THP-1 cells or mouse macrophages were incubated for 30 min at 37°C with fluorescein isothiocyanate (FITC)-conjugated monoclonal antibodies (mAbs) against CCR7 in PBS. The cells were washed twice with PBS, and their fluorescence was assessed by flow cytometry.

### Induction of LPS-induced Endotoxin Shock

Male 6-week-old C57BL/6 mice (20.1±0.77 g) were intraperitoneally (i.p.) injected with 400 µg of LPS from *Escherichia coli* serotype 055:B5 (Sigma-Aldrich). Several hours after the injection, concentrations of IL-1ß, IL-6, IL-12 and TNF-alpha in serum and peritoneal lavage fluid were measured by ELISA. The M1-type polarization of spleen and peritoneal macrophages was analyzed. The mice were treated and handled according to the Guide Principles for the Care and Use of Laboratory Animals of the Japanese Pharmacological Society and with the approval of Tokyo University of Science’s Institutional Animal Care and Use Committee (Permit Number: S12017).

### Statistics

Results are expressed as mean ± SE. The statistical significance of differences between control and other groups was calculated by using Dunnett’s test with the Instat version 3.0 statistical package (GraphPad Software). The criterion of significance was set at P<0.05.

## Results

### LPS-induced ATP Release via Exocytosis from THP-1 Cells

**Figure 1:**
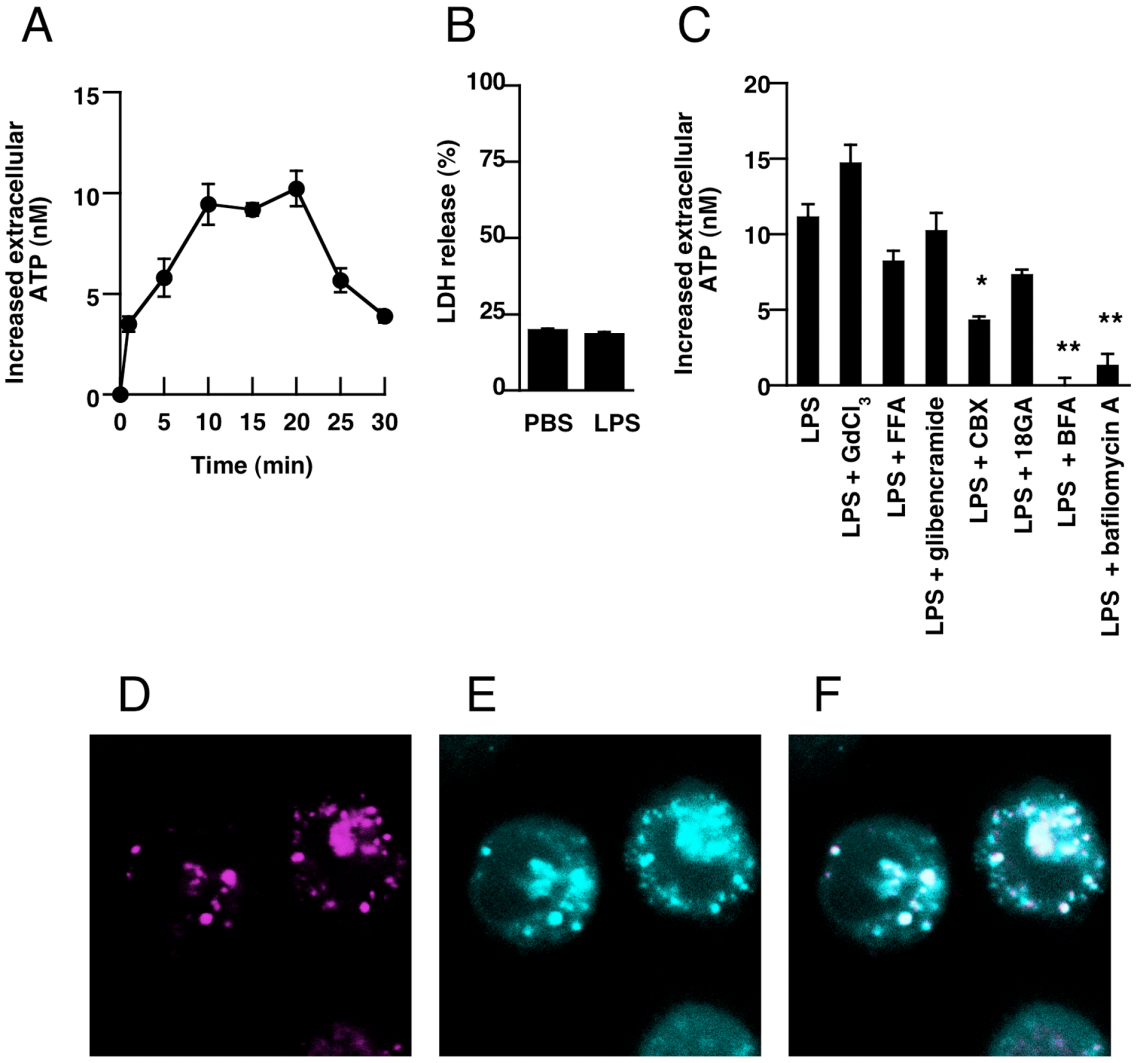
LPS-induced ATP release from THP-1 cells. (**A**) Cells were stimulated with LPS (10 µg/mL) and incubated for the indicated times, then the concentration of ATP in the culture medium was measured as described in Materials and Methods. (**B**) Cells were incubated with PBS or LPS for 30 min. At the end of incubation, supernatants were collected and LDH content was measured. The release of LDH is expressed as a percentage of total content determined by lysing an equal number of cells with 1% Triton X-100. (**C**) Cells were pretreated with GdCl_3_ (100 µM), FFA (50 µM), glibenclamide (100 µM), CBX (50 µM), 18GA (50 µM), BFA (10 µM) or bafilomycin A (50 nM) for 30 min. At 10 min after LPS stimulation, supernatants were collected and ATP contents were measured. (**D–F**) Cells were stained with MANT-ATP (50 µM, cyan, panel C) and quinacrine dihydrochloride (5 µM, magenta, panel D) for 1 h at 37°C. Then, stained cells were analyzed using a confocal laser scanning microscopy. The merged image is shown in panel E. Each value represents the mean ± SE (n = 4–8). Significant differences between the indicated groups and control group (LPS alone) are indicated by *(p<0.05) and **(P<0.01), respectively.

THP-1 human monocytes were primed with LPS and IFN-gamma for 24 h to induce differentiation into macrophages. Primed THP-1 cells were stimulated with 10 µg/mL of LPS, and the extracellular concentration of ATP was measured. As shown in Figure 1A, the extracellular concentration of ATP was increased soon after stimulation with LPS and peaked at 10 to 20 min. In this method, ATP released from cells is diluted in the culture medium and is rapidly metabolized by ecto-nucleotidases on the plasma membrane. Therefore, the detected concentration of ATP must be much lower than the real concentration at the cell surface. The peri-cellular concentration of ATP just after ATP release would be much higher than detected concentration of ATP in medium. We confirmed that treatment with LPS did not induce cell death within 30 min (Figure 1B), suggesting that LPS-induced ATP release would not be dependent on leakage of ATP from dead cells. In subsequent pharmacological experiments, we measured extracellular ATP at 10 min after stimulation with LPS.

To identify the pathway of the ATP release, we examined the effects of several inhibitors. As shown in Figure 1C, pretreatment with CBX (inhibitor of pannexin hemichannel) blocked the release of ATP after LPS stimulation. Moreover, pretreatment with bafilomycin A (inhibitor of vesicular H^+^-ATPase) and BFA (inhibitor of vesicular trafficking), which are inhibitors of vesicular exocytosis of ATP, significantly blocked the release of ATP after LPS stimulation. However, pretreatment with 18GA (connexin hemichannel inhibitor), FFA (anion transporter inhibitor), glibenclamide (anion transporter inhibitor), or GdCl_3_ (maxi-anion channel inhibitor) did not block the ATP release. These results indicated that the release of ATP after LPS stimulation is regulated by plural mechanisms, including pannexin hemichannels and vesicular exocytosis.

To look for ATP-enriched vesicles in THP-1 cells, we stained intracellular ATP with the fluorescent ATP indicator MANT-ATP. Fluorescent vesicles were observed in the cytoplasm of primed THP-1 cells (Figure 1D). Protons are accumulated in ATP-enriched vesicles by vacuolar proton-ATPase, forming an electrochemical gradient of protons across the membrane that serves as the driving force for ATP accumulation. Staining with a low pH-sensitive fluorescent probe, quinacrine dihydrochloride, visualized the same population of vesicles as that stained by MANT-ATP (Figure 1E, F), indicating that ATP-enriched vesicles are present in primed THP-1 cells.

**Figure 2:**
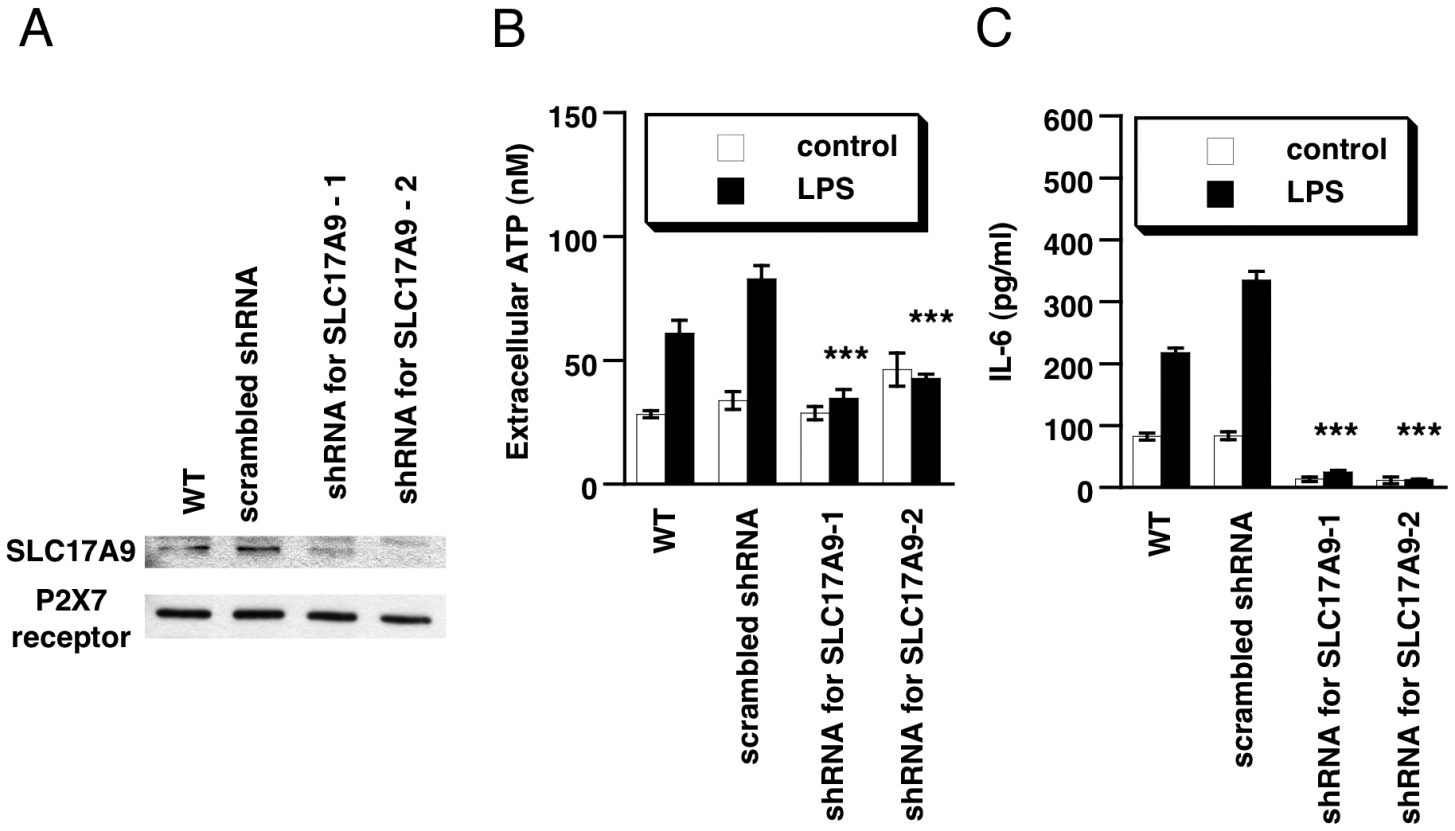
SLC17A9 is involved in LPS-induced ATP release and IL-6 production in THP-1 cells. (**A**) THP-1 cells were transfected with 2 µg of two different shRNAs targeting SLC17A9 (No. 1 or No. 2) or scramble shRNA. Seventy-two hours after transfection, cellular membrane proteins were extracted and the expression of SLC17A9 was detected by immunoblotting as described in Materials and Methods. (**B**) THP-1 cells transfected with two different shRNAs targeting SLC17A9 or the negative control shRNA were stimulated with LPS. At 10 min after LPS stimulation, the supernatants were collected and ATP content was measured (n = 8–12). (**C**) The shRNA-transfected cells were incubated with LPS for 24 h, and then the concentration of IL-6 in culture medium was measured by ELISA (n = 10–14). Each value represents the mean ± SE. Significant differences between the control group (scramble) and the indicated group are indicated by ***(p<0.001).

Next, to investigate whether exocytosis is involved in LPS-induced ATP release from THP-1 cells, we silenced the expression of SLC17A9 using two different shRNAs. We confirmed that mRNA and protein expression of SLC17A9 were suppressed by transfection with SLC17A9-shRNA (Figure 2A). The decreased SLC17A9 expression resulted in suppression of ATP release compared with that of scramble shRNA-transfected cells, in response to LPS stimulation (Figure 2B). Furthermore, we investigated the involvement of vesicular exocytosis of ATP in LPS-induced IL-6 production. LPS-induced IL-6 production was significantly suppressed in SLC17A9-knockdown cells (Figure 2C). These results indicated that LPS induces ATP release from macrophages via vesicular exocytosis, and this process is involved in LPS-induced IL-6 production.

### Role of P2Y11 Receptor in LPS-induced M1-type Polarization and IL-6 Production in Macrophages

**Figure 3:**
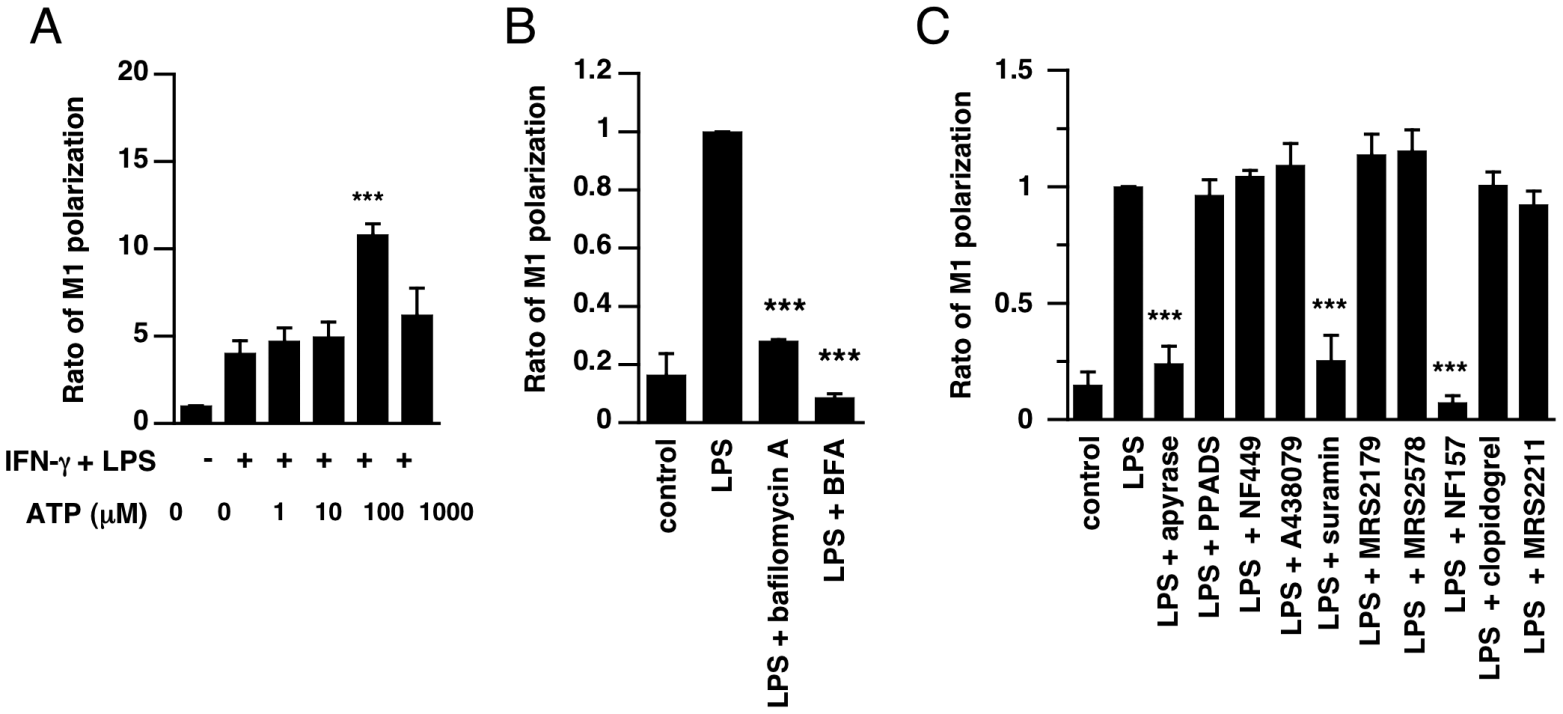
Extracellular ATP contributes to M1 macrophage polarization via activation of P2 receptors. (**A–C**) THP-1 cells were cultured for 24 h in medium or in medium supplemented with LPS and IFN-gamma in the absence or presence of ATP (**A**), or bafilomycin A (50 nM), BFA (10 µM) (**B**), apyrase (25 U/mL), PPADS (100 µM), A438079 (100 µM), suramin (100 µM), NF449 (10 µM), MRS2179 (100 µM), MRS2578 (10 µM), NF157 (50 µM), clopidogrel (30 µM), or MRS2211 (100 µM) (**C**). Expression of M1 macrophage marker CCR7 was measured by flow cytometry. The supernatants were assayed for IL-6 (n = 8–10). Each value represents the mean ± SE. A significant difference between the control group (non-treated or LPS alone) and a test group is indicated by ***(p<0.001).

M1 polarization supports resistance to intracellular bacteria and control of the acute phase of infection. First, we investigated how extracellular ATP affects M1-type macrophage polarization. When THP-1 cells were stimulated with LPS and IFN-gamma, THP-1 cells were polarized into M1-type macrophages. Addition of ATP (100 µM) significantly facilitated M1-polarization of THP-1 cells (Figure 3A), indicating that extracellular ATP contributes to effective polarization of M1-type macrophages.

To investigate the involvement of ATP release via exocytosis in M1-polarization, we examined the effect of bafilomycin A or BFA on M1-polarization of THP-1 cells. Pretreatment with these inhibitors significantly blocked M1-polarization (Figure 3B), suggesting an involvement of ATP release via exocytosis in M1-polarization of THP-1 cells.

Next, we examined the effects of P2 receptor antagonists on M1 macrophage polarization. It is reported that macrophages express P2 receptors such as P2X1, P2X7, P2Y1, P2Y2, P2Y6 and P2Y11 receptors [30], [31]. Pretreatment with apyrase (ecto-nucleotidase) significantly suppressed M1 macrophage polarization, suggesting the involvement of extracellular ATP. Although pretreatment with PPADS (P2X antagonist), A438079 (P2X7 antagonist), NF449 (P2X1 antagonist), MRS2179 (P2Y1 antagonist), MRS2578 (P2Y6 antagonist), clopidogrel (P2Y12 antagonist), or MRS2211 (P2Y13 antagonist) did not affect induction of M1 macrophage polarization, a broad P2Y receptor antagonist, suramin, significantly blocked induction of M1-type macrophage polarization (Figure 3C). Furthermore, a highly selective P2Y11 receptor antagonist, NF157, also significantly blocked the induction, indicating the involvement of P2Y11 receptor in M1 macrophage polarization.

**Figure 4:**
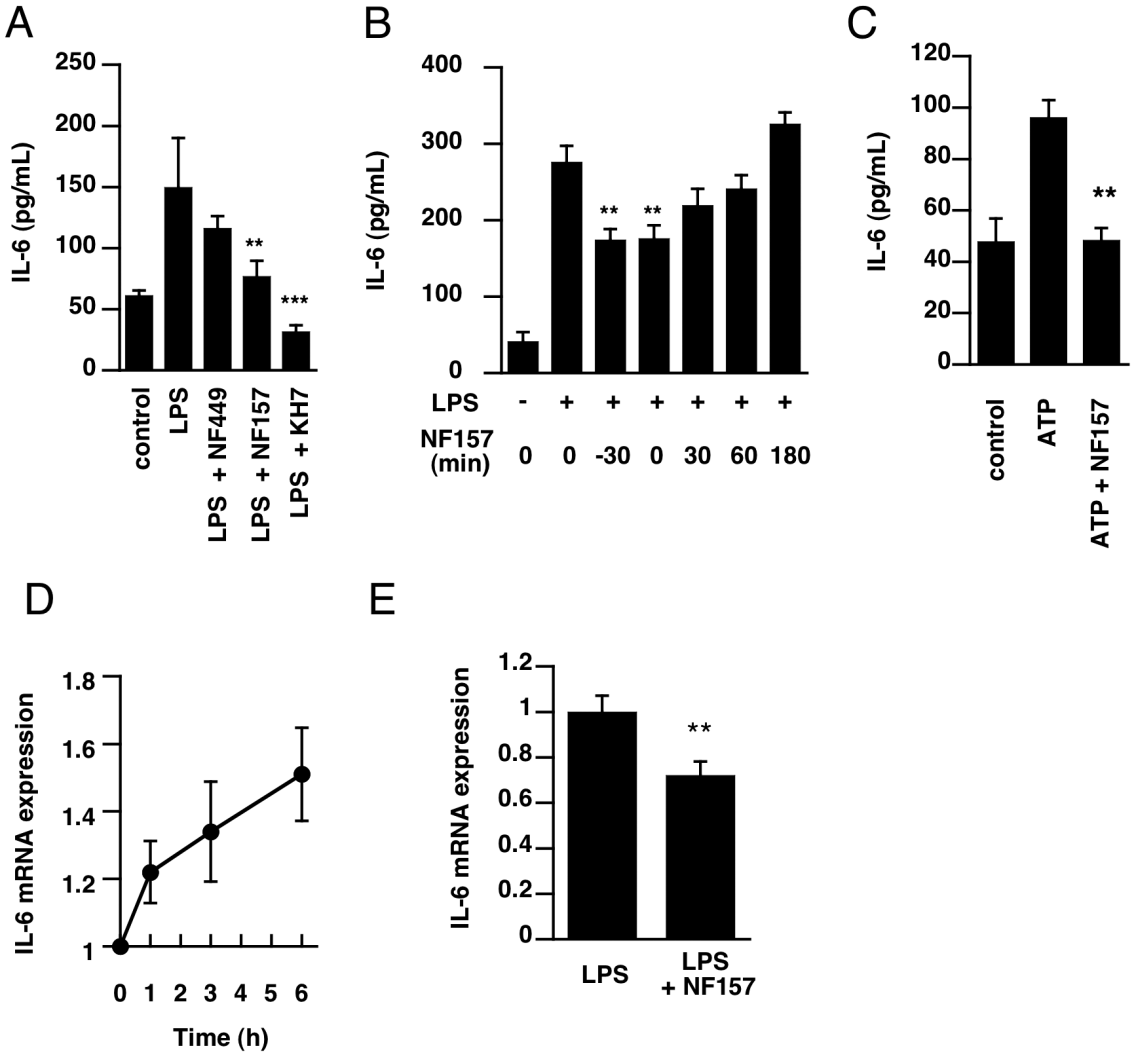
Effect of P2Y11 receptor antagonist on LPS-induced IL-6 production in THP-1 cells. (**A**) THP-1 cells were pretreated with NF449 (10 µM), NF157 (50 µM), or KH7 (100 µM) for 30 min. (**B**) Treatment with NF157 was initiated 30 min before to 180 min after the treatment with LPS (10 µg/mL). At 24 h after LPS stimulation, supernatants were collected and the concentration of IL-6 was measured by ELISA. (**C**) Cells were pre-treated with NF157 for 30 min, and then incubated with 100 µM ATP for 24 h. Supernatants were collected and the concentration of IL-6 was measured by ELISA. (**D–E**) Cells were incubated with LPS (10 µg/mL) for 1–6 h (**D**). Cells were pre-incubated with NF157, and incubated with LPS for 6 h (**E**). At the end of incubation, mRNA level of IL-6 was determined by RT^2^-PCR as described under *Materials and Methods*. Each value represents the mean ± SE (n = 4–11). Significant differences between a test group and control group (LPS or ATP alone) are indicated by **(p<0.01) and ***(p<0.001), respectively.

We also examined the effect of NF157 on LPS-induced IL-6 production. Pretreatment with NF157 significantly blocked the production of IL-6 in THP-1 cells (Figure 4A). Although NF157 can inhibit not only P2Y11, but also P2X1 receptor, pretreatment with selective P2X1 antagonist NF449 was ineffective (Figure 4A), suggesting that the inhibitory effect of NF-157 on IL-6 production is dependent on the blockade of P2Y11 receptor in THP-1 cells.

To further investigate the contribution of P2Y11 receptor to IL-6 production, we examined the effect of adenylate cyclase inhibitor KH7, because P2Y11 receptor is coupled with Gs-protein, which activates adenylate cyclase [32]. Although P2Y11 receptor is coupled with both Gs-protein and Gq-protein, it is the only Gs-coupled P2 receptor among the P2 receptor subtypes [32]. Pretreatment with KH7 completely blocked IL-6 production, indicating that increase of cAMP is critical for IL-6 production (Figure 4A). The inhibitory effect of NF157 on LPS-induced IL-6 production disappeared when NF157 treatment was done 30 min after LPS treatment, indicating that P2Y11 receptor is activated within 30 min after LPS stimulation (Figure 4B). Further, treatment with 100 µM ATP also causes IL-6 production, and ATP-induced IL-6 production was significantly inhibited by pretreatment with NF157 (Figure 4C). These results suggest that ATP-induced activation of P2Y11 receptor would be involved in LPS-induced IL-6 production.

We investigated the effect of NF157 on induction of IL-6 mRNA expression after LPS stimulation. The level of IL-6 mRNA was increased from 1 h to 6 h after LPS-stimulation (Figure 4D). The increase of IL-6 mRNA at 6 h was partially suppressed by pretreatment with NF157 (Figure 4E), suggesting that NF157 would suppress IL-6 production via both transcriptional and post-transcriptional levels.

**Figure 5:**
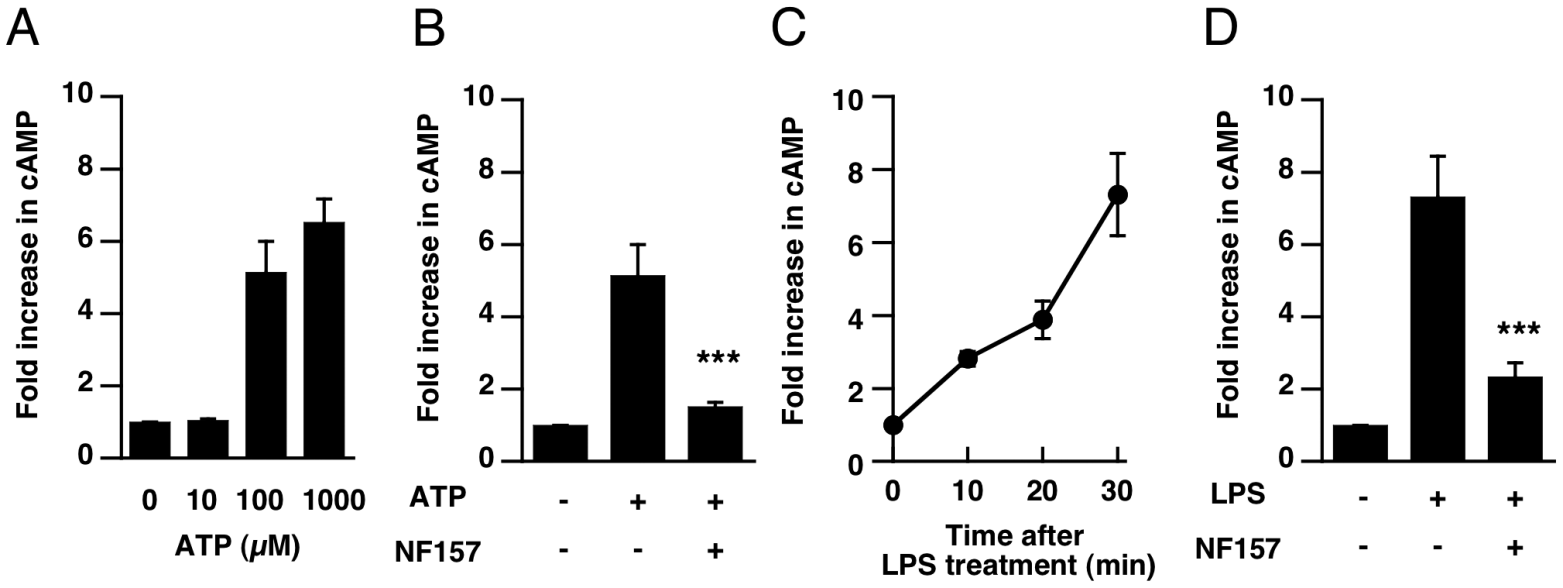
P2Y11 antagonist NF157 blocked ATP or LPS-induced increase of cAMP levels in THP-1 cells. (A) THP-1 cells were incubated with 10–100 µM of ATP for 10 min. (B) Cells were pre-incubated with NF157 (50 µM) for 30 min, and then incubated with 100 µM ATP for 10 min. (C) Cells were incubated with LPS (10 µg/mL) for indicated times. (D) Cells were pr)e-incubated with NF157 for 30 min, and then incubated with LPS. Intracellular cAMP level was measured as described in *Materials and Methods*. Data is expressed as fold increase in cAMP compared with control. Each value represents the mean ± SE (n = 4). Significant differences between the positive control group (ATP or LPS alone) and the indicated group are indicated by ***(p<0.001).

To confirm ATP-induced activation of P2Y11 receptor in THP-1 cells, we measured a change in cAMP level after treatment with 10–1000 µM of ATP because P2Y11 receptor is Gs-protein coupled receptor [32]. At 10 minutes after treatment with ATP, cAMP level was significantly increased by 100 µM or 1 mM of ATP in THP-1 cells (Figure 5A). The increase of cAMP was inhibited by pretreatment with NF157 (Figure 5B), suggesting ATP-induced activation of P2Y11 receptor in THP-1 cells. Further, we investigated the activation of P2Y11 receptor after LPS stimulation in THP-1 cells. Treatment with LPS caused cAMP elevation in a time-dependent manner (Figure 5C), and the increase was blocked by NF157 (Figure 5D), indicating the activation of P2Y11 receptor after LPS stimulation.

**Figure 6:**
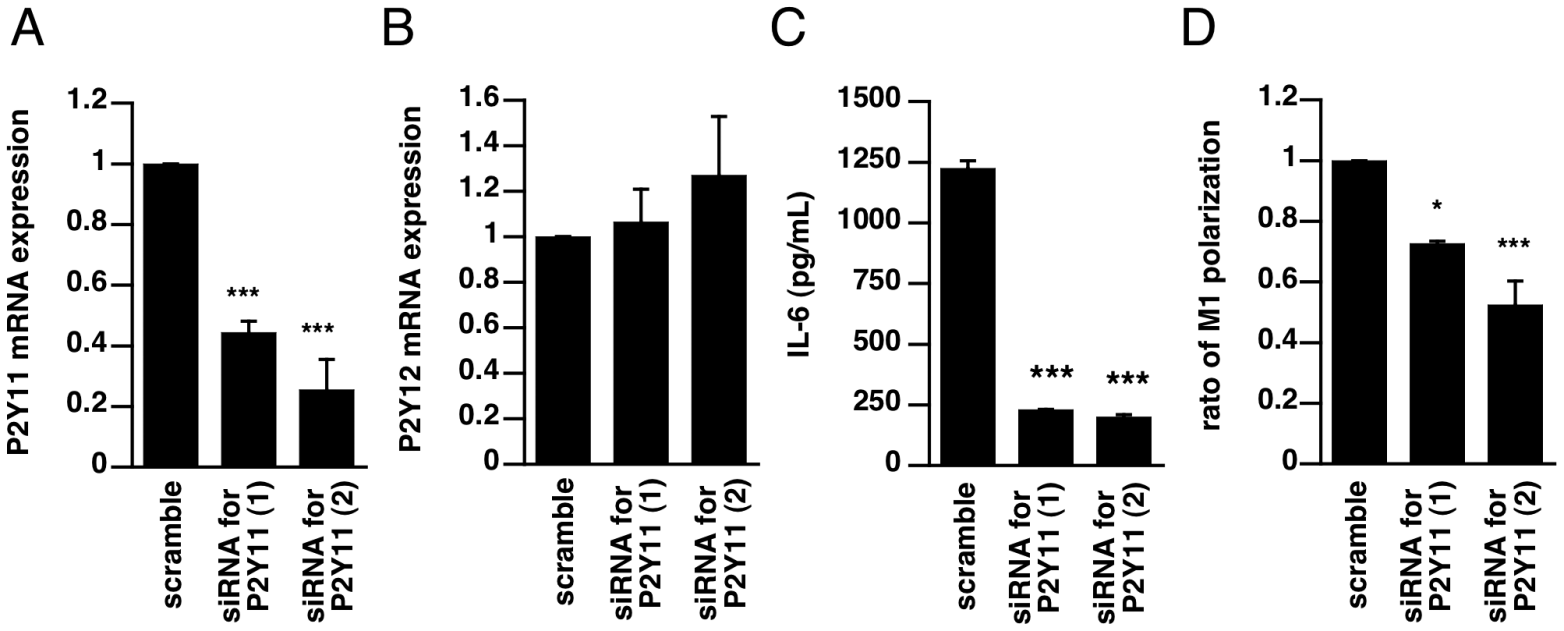
P2Y11 receptor is involved in IL-6 production and M1 macrophage polarization in THP-1 cells. (**A–B**) THP-1 cells were transfected with 3 µg of shRNA targeting P2Y11 (No.1 or No.2) or scramble shRNA. Twenty-four hours after transfection, total RNA was extracted and P2Y11 or P2Y12 gene expression was examined by assessing mRNA levels using real-time PCR. (**C**, **D**) Cells were transfected with siRNA targeting P2Y11 (No.1 or No.2) or scramble siRNA and incubated for 72 h. (**C**) The transfected cells were stimulated with LPS (10 µg/mL) and incubated for 24 h, and then the concentration of IL-6 in culture medium was measured by ELISA. (**D**) The transfected cells were cultured for 24 h in medium or in medium supplemented with LPS and IFN-gamma. Expression of M1 macrophage marker CCR7 was measured by flow cytometry. Each value represents the mean ± SE (n = 4–8). Significant differences between a test group and control group (scramble) are indicated by *(p<0.05) and ***(p<0.001), respectively.

To confirm the involvement of P2Y11 receptor in IL-6 production and M1-polarization, P2Y11 receptor in THP-1 cells was knocked down by transfection with siRNA. The mRNA expression of P2Y11 receptor was decreased to 44.4% (siRNA-1) or 25.4% (siRNA-2) (Figure 6A). We confirmed that these transfections did not affect the expression of other receptors by detecting an expression of P2Y12 receptor mRNA (Figure 6B). Treatment of P2Y11-knockdown THP-1 cells with LPS failed to induce IL-6 production (Figure 6C) or M1 polarization (Figure 6D). These results indicate that P2Y11 receptor plays a major role in LPS-induced activation of macrophages.

### P2Y11 Antagonist NF157 Attenuates the Elevation of Inflammatory Cytokines in Peritoneal Fluid and Serum of LPS-induced Septic Mice

We next investigated the effect of NF157 on activation of macrophages *in vivo*. However, among mammals, the expression of P2Y11 receptors has been found only in humans and canines, although the existence of a receptor with properties like those of the P2Y11 receptor in mice and rats has been suggested [33], [34], [35]. Since some papers have reported that NF157 inhibits murine P2Y11 like receptors [34], [35], we examined the effect of NF157 on P2Y11 like receptor in mouse macrophages. Treatment with 100 µM ATP caused increase of cAMP level, and NF157 partially suppressed ATP-induced cAMP elevation in mouse macrophage RAW264.7 cells (Figure S1). We examined the effect of NF157 on LPS-induced IL-6 production by isolated peritoneal macrophages. NF157, but not NF449, partially suppressed LPS- or ATP-induced IL-6 production from peritoneal macrophages (Figure S2). These results suggest that NF157 can suppress an activation of mouse P2Y11 like receptor partially.

**Figure 7:**
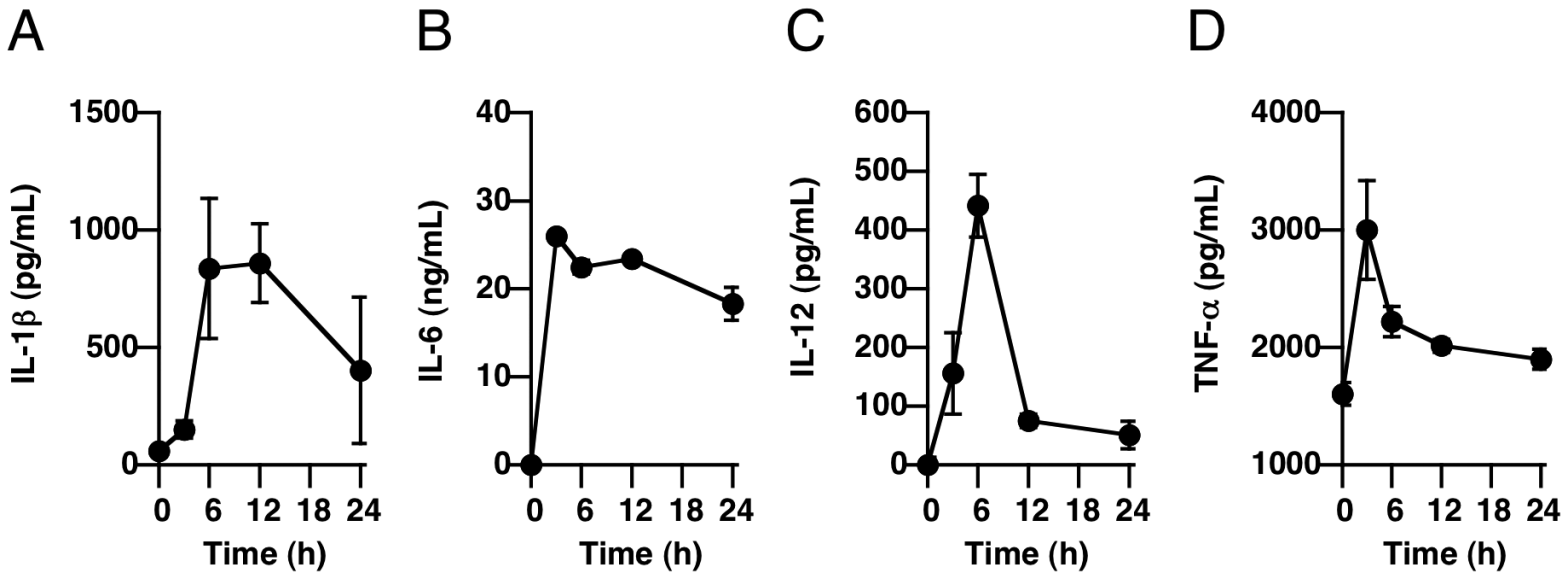
LPS treatment induced increased serum levels of cytokines. (**A**–**D**) Male C57BL/6 mice at 6 weeks of age were given LPS (400 µg/head i.p.), and blood samples were collected at 3, 6, 12, and 24 h after LPS injection. Serum levels of IL-1ß, IL-6, IL-12 and TNF-alpha were determined as described in *Materials and Methods*.

It is well known that high doses of LPS induce excessive immune activation in vivo, and this resembles sepsis. We confirmed the elevation of pro-inflammatory cytokine levels in serum after administration of LPS to mice. The concentrations of IL-1ß, IL-6, IL-12, and TNF-alpha in serum were significantly increased at 3–24 h after administration of LPS (Figure 7A–D).

**Figure 8:**
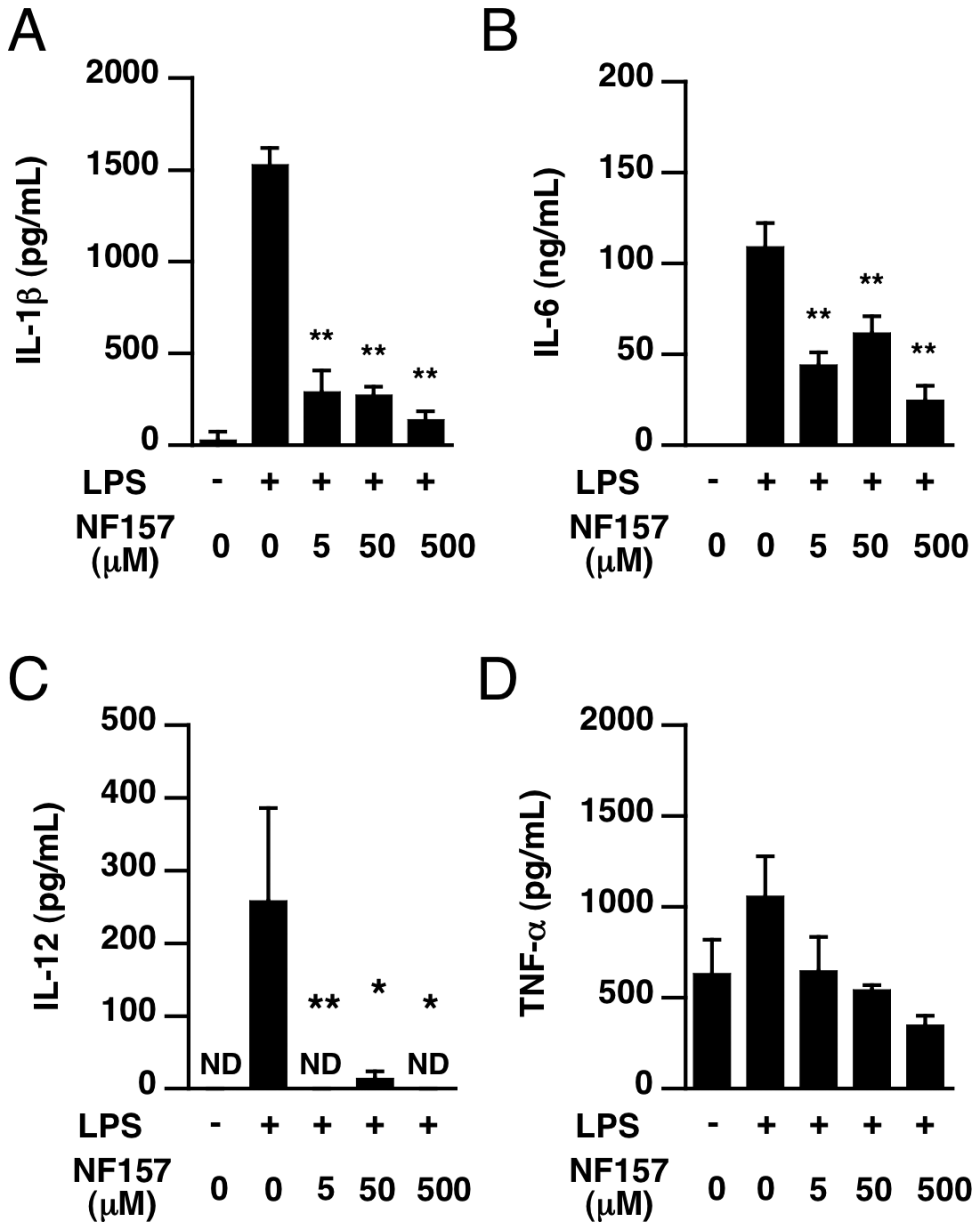
P2Y11 antagonist NF157 suppressed the increase in serum levels of cytokines in LPS-treated mice. (**A**–**D**) Male C57BL/6 mice at 6 weeks of age were given LPS (400 µg/head i.p.). NF157 (100 µL) doses of 5, 50 and 500 µM were administered intraperitoneally at 2 h before LPS was injected. Blood samples were collected at 6 h after LPS injection. Serum levels of IL-1ß, IL-6, IL-12 and TNF-alpha were determined as described in *Materials and Methods*. Each value represents the mean ± SE (n = 4). Significant differences between a test group and control group (LPS alone) are indicated by *(p<0.05) and **(p<0.01), respectively.

To investigate whether P2Y11 receptor antagonist NF157 suppresses LPS-induced overproduction of cytokines *in vivo*, NF157 (100 µl at indicated concentration/head) was administered i.p. at 2 h before LPS injection. The cytokine levels in serum were measured at 6 h after treatment with LPS. As shown in Figure 8, treatment with NF157 significantly suppressed the increases of TNF-alpha, IL-1ß, IL-6 and IL-12 in serum. The inhibitory effects were dose-dependent, with a maximal effect at 500 µM. Even the lowest dose of NF157 examined (5 µM) was sufficient to suppress the production of cytokines. On the other hand, administration of P2X1 antagonist NF449 with LPS did not suppress increase of IL-1ß, IL-6, IL-12 and TNF-alpha in serum (Figure S3).

**Figure 9:**
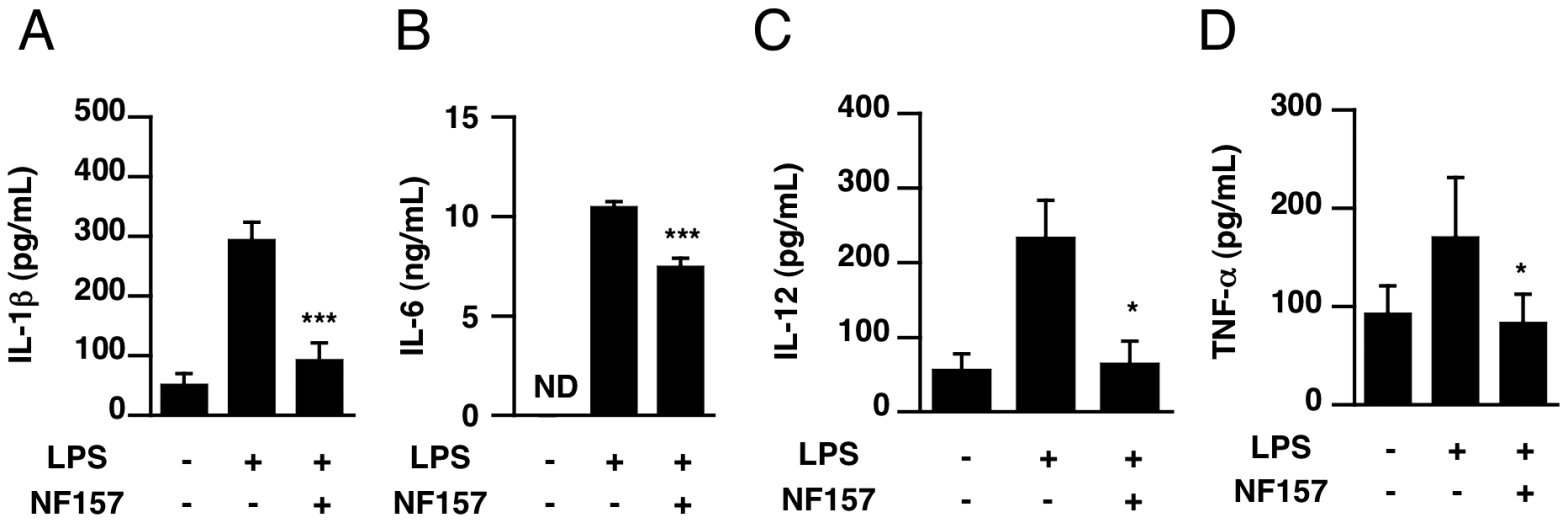
P2Y11 antagonist NF157 blocked the increase in peritoneal levels of cytokines in LPS-treated mice. (A –**D**) Male C57BL/6 mice were injected intraperitoneally with LPS (400 µg/head i.p.). NF157 (100 µL of 500 µM) was administered i.p. at 2 h before LPS was injected. Peritoneal lavage fluid was collected 6 h after LPS injection for measurement of IL-1ß, IL-6, IL-12 and TNF-alpha. Each value represents the mean ± SE (n = 4). Significant differences between a test group and control group (LPS alone) are indicated by *(p<0.05) and ***(p<0.001), respectively.

**Figure 10:**
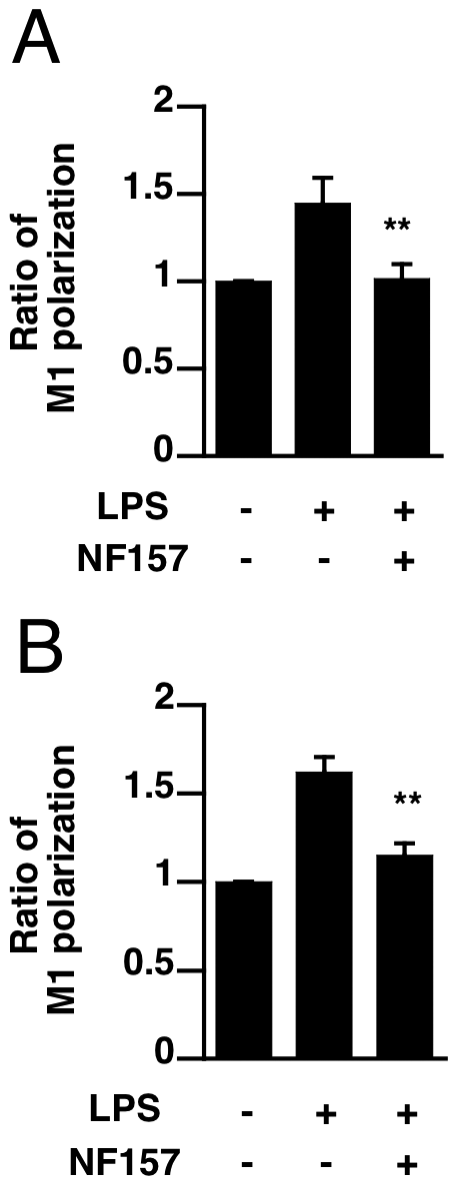
NF157 blocked M1 polarization of macrophages in LPS-treated mice. (**A**, **B**) Peritoneal and splenic macrophages were isolated from mice 24 h after LPS injection, and analyzed by flow cytometry for the expression of CCR7. Each value represents the mean ± SE. A significant difference between the positive control group (LPS alone) and the indicated group is indicated by **(p<0.01).

We also measured the cytokine levels in peritoneal fluid and M1-type polarization of peritoneal and splenic macrophages. Administration of NF157 with LPS inhibited the LPS-induced increases of cytokine levels (IL-1ß, IL-6, IL-12 and TNF-alpha) in peritoneal lavage fluid at 6 h after LPS treatment; these increases would mainly reflect release from peritoneal macrophages stimulated by LPS (Figure 9A–D). The To determine whether NF157 treatment modulates LPS-induced M1 macrophage polarization *in vivo*, peritoneal and splenic macrophages were isolated from mice at 24 h after LPS injection, and the expression of CCR7 was examined. As shown in Figure 10A–B, treatment with NF157 at 2 h before LPS injection effectively blocked LPS-induced M1-type polarization of peritoneal and splenic macrophages. These results suggest that treatment with NF157 might be effective for suppression of innate inflammatory response in polymicrobial septic shock.

**Figure 11:**
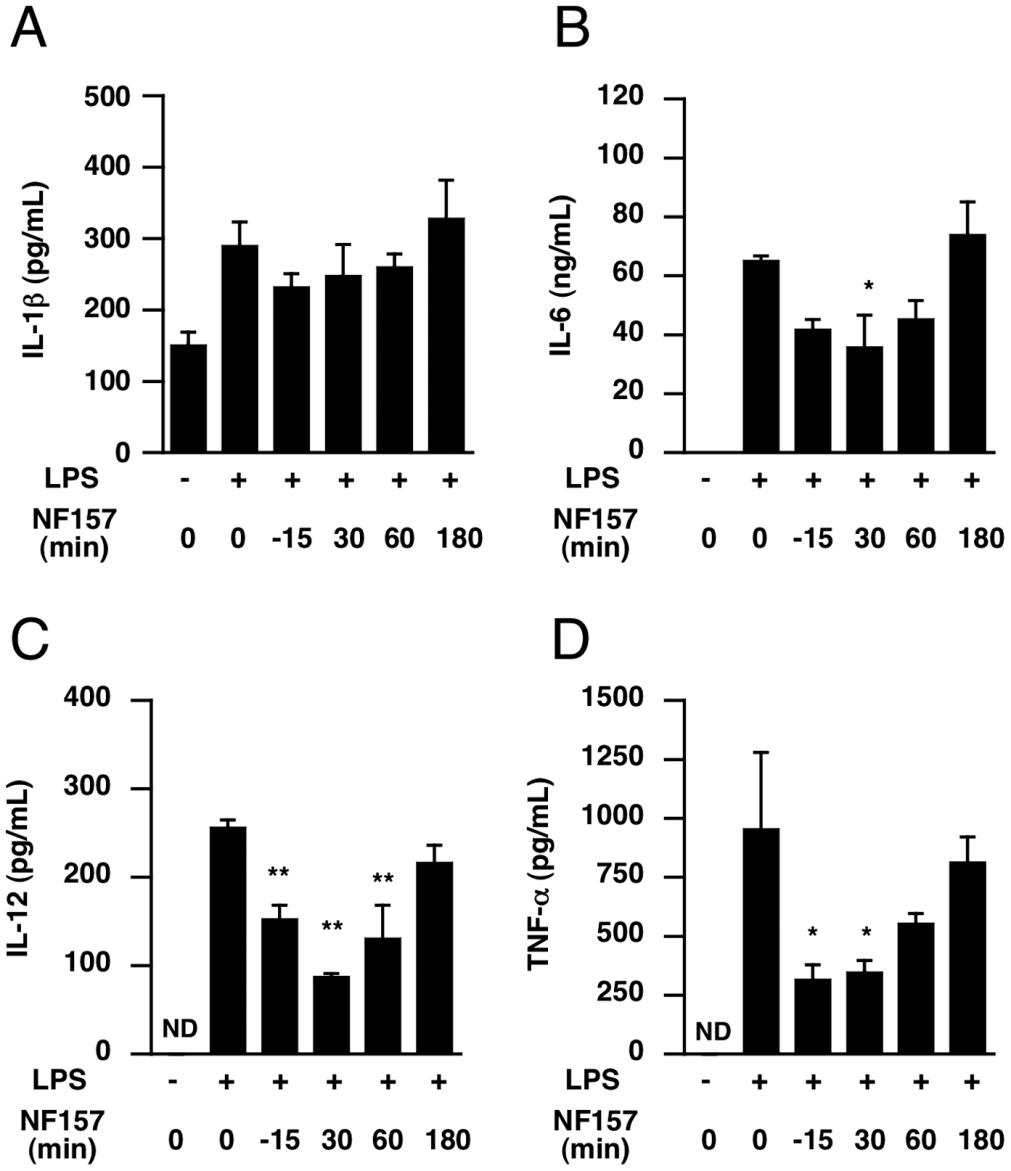
Suppressive effect of post-treatment with NF157 on serum levels of cytokines in LPS-treated mice. (**A**–**D**) Male C57BL/6 mice at 6 weeks of age were given LPS (400 µg/head i.p.). Treatment with NF157 was initiated 15 min before to 180 min after the onset of LPS injection. Blood samples were collected at 6 h after LPS injection. Serum levels of IL-1ß, IL-6, IL-12 and TNF-alpha were determined as described in *Materials and Methods*. Each value represents the mean ± SE (n = 4). Significant differences between a test group and control group (LPS alone) are indicated by *(p<0.05) and **(p<0.01), respectively.

Finally, we assessed the inhibitory effect of post-treatment with NF157 on the onset of endotoxemia. NF157 was administered at 30–180 min after LPS, and serum cytokine levels were measured. Elevation of IL-6, IL-12, and TNF-alpha, but not IL-1ß, was significantly attenuated even when mice were post-treated with NF157 at 30–60 min after administration of LPS, indicating that treatment with NF157 might be effective in blocking the onset of endotoxemia even after activation of macrophages by LPS (Figure 11A–D).

## Discussion

Since activation of macrophages contributes to severe inflammation, such as occurs in sepsis, it is important to understand the process of macrophage activation. In this study, we investigated the involvement of extracellular ATP-mediated purinergic signaling in macrophage activation. The most striking conclusion of this work is that endogenous ATP, released via exocytosis, activates P2Y11 receptor as a crucial second signal for inducing M1 polarization of macrophages and production of pro-inflammatory cytokine IL-6. In accordance with this idea, we found that the P2Y11 antagonist NF157 effectively suppressed the LPS-induced innate inflammatory response in mice. This report is the first to identify vesicular exocytotic ATP release from macrophages, and we also established that this exocytotic ATP release plays a major role in macrophage activation and IL-6 production. Considering the results of our recent study on the involvement of vesicular exocytosis in T cell activation [28], autocrine regulation via exocytosis of ATP appears likely to play an important role in effective activation of immune cells. This hypothesis is also supported by our finding that ATP facilitated M1-type polarization of macrophages.

Before the identification of SLC17A9 as a vesicular nucleotide transporter [25], it had been difficult to establish the involvement of exocytosis in ATP release caused by various stimuli. Recently, by using transfecting human T cells with shRNA for SLC17A9, we have demonstrated the involvement of exocytosis in ATP release caused by activation of T cell receptor [28], as well as ATP release from lung cancer cells caused by TGF-ß1 [29]. Here, we used the same approach to show that vesicular exocytosis is also involved in LPS-induced macrophage activation. It has been reported that pannexin hemichannels and maxi-anion channels are also involved in ATP release during activation of T cells [36]. In the present study too, LPS-induced ATP release from macrophages was found to be reduced by treatment with an inhibitor of pannexin hemichannels. Thus, multiple pathways may contribute to ATP release from LPS-treated macrophages. Nevertheless, our data indicated that abrogation of exocytotic ATP release caused significant suppression of IL-6 production of macrophages, suggesting that activation of P2 receptors by exocytotically released ATP plays a major role in mediating IL-6 production induced by LPS. Since LPS-induced ATP release in mouse peritoneal fluid has been confirmed *in vivo* [37], it is very likely that ATP release has a substantial role in the LPS-induced innate immune response.

Our data indicate that extracellular ATP enhances LPS- and IFN-gamma-induced M1 polarization. In particular, treatment with 100 µM ATP, but not 1 mM ATP, caused facilitation of M1-type polarization. It is consistent with the data indicating cAMP elevation by 100 µM ATP. Though cAMP elevation was also observed by treatment with 1 mM ATP, the enhancement of M1 polarization was not observed in 1 mM ATP-treated cells. The reason why 1 mM ATP did not enhance M1 polarization would be that high dose of ATP negatively regulate M1 polarization via pyrophosphates, which is not dependent on purinergic receptor [38].

Since treatment with inhibitors of the exocytosis of ATP or ecto-nucleotidase significantly inhibited M1-type polarization, ATP release via exocytosis would play an important role in M1-type polarization. Treatment with P2Y11 receptor antagonist NF157 and knockdown of P2Y11 receptor both caused significant suppression of M1 polarization, indicating the involvement of P2Y11 receptor in the M1-type polarization. Since P2Y11 receptor is involved in differentiation of various cell types, such as monocyte-derived dendritic cells [39], the involvement of P2Y11 receptor in macrophage differentiation seems reasonable. Blockade of M1-type polarization must cause suppression of various inflammatory events associated with macrophages and other immune cells, which are stimulated by cytokines produced by M1-macrophages. Indeed, the LPS-induced increase of serum concentrations of inflammatory cytokines, which is a result of systemic immune response, was significantly suppressed by pretreatment of mice with NF157.

Treatment with ATP, even without LPS, induced IL-6 production, suggesting an essential role of extracellular ATP in IL-6 production in macrophages. LPS-induced IL-6 production was significantly suppressed by knockdown of the P2Y11 receptor. Since IL-6 production was also significantly suppressed by treatment with adenylate cyclase inhibitor, increase of cAMP appears to play a critical role in IL-6 production. The only P2 receptor coupled via Gs to adenylate cyclase is the P2Y11 receptor [32], so it is reasonable that induction of cAMP is involved in P2Y11-mediated IL-6 production. We also confirmed treatment with ATP causes cAMP elevation in THP-1 cells, and it was blocked by NF157, suggesting that extracellular ATP can induce the activation of P2Y11 receptor in THP-1 cells. Inhibition of IL-6 production was not observed *in vitro* when NF157 was applied 30 min after LPS, suggesting that activation of P2Y11 receptor occurs within 30 min after LPS stimulation to induce IL-6 production. It is consistent with the data indicating that LPS caused cAMP elevation within 30 min, which was inhibited by NF157. Since elevation of IL-6 level in serum is observed at an early phase of sepsis, blockade of P2Y11 receptor might be effective for suppression of IL-6 over-production in the early phase of sepsis. It has been reported that Gs-protein-coupled rat P2Y11-like receptor is involved in transcriptional regulation of IL-6 in bile duct epithelia [40], and this is consistent with our findings. Indeed, we found that injection of LPS into the peritoneal cavity of mice caused elevation of inflammatory cytokine levels in serum, and pretreatment with NF157 significantly suppressed the elevation of serum and peritoneal cytokine levels in these mice. Moreover, LPS-induced M1-polarization of splenic and peritoneal macrophages was suppressed by treatment with NF157. All these results suggest that blockade of P2Y11 by NF157 is effective for suppression of macrophage activation induced by LPS *in vivo*. Interestingly, this inhibitory effect was observed even at 30 min after LPS treatment, presumably before all the macrophages are activated.

Treatment with NF157 caused significant suppression of not only IL-6, but also IL-1ß, IL-12, and TNF-alpha elevation in serum and peritoneal fluid, presumably as a result of blockade of M1-polarization of macrophages. The effect of NF157 on peritoneal cytokine production may represent a direct effect on LPS-stimulated peritoneal macrophages. On the other hand, the elevation of cytokine levels in serum would be due to LPS-induced systemic immune response. In the peritoneal fluid, NF157 significantly diminished the production of IL-1ß, IL-12, TNF-alpha, but only partially suppressed IL-6 production. The inhibitory effect of NF157 on IL-6 production was lower in isolated mouse peritoneal macrophages than in human monocyte THP-1 cells, which is consistent with a partial inhibition of P2Y11 like receptor by NF157 in mouse macrophages. It has also been reported that P2Y11 agonist stimulates release of IL-8 from human monocyte-derived dendritic cells [41]. Overall, because NF157 blocks M1-type polarization of peritoneal and spleen macrophages, it seems reasonable that NF157 would effectively inhibit the whole-body immune response in humans. In any case, human P2Y11 receptor and mouse P2Y11 like receptor appear to have important roles in immune regulation.

P2X7 receptor is well known to induce IL-1ß release by activating caspase-1, which convert pro-IL-1ß to mature IL-1ß. On the other hand, blockade of P2Y11 receptor causes suppression of M1-type polarization, which would block pro-IL-1ß production. Thus, the mechanism of the blockade of IL-1ß release might be different between P2X7 receptor antagonists and P2Y11 receptor antagonists.

In conclusion, we have identified a novel mechanism of macrophage activation, which is mediated by autocrine regulation via exocytotic ATP release and activation of P2Y11 receptor. Our results suggest that P2Y11 receptor antagonists can block the activation of macrophages by LPS both *in vitro* and *in vivo*, and suppress the elevation of inflammatory cytokines, but further studies will be needed to investigate the effect of P2Y11 receptor antagonists on the pathological changes that occur in sepsis by using cecal ligation puncture model, which is a chronic sepsis model, or in other inflammatory diseases. Though blockade of mouse P2Y11 like receptor by NF157 was partially, significant suppression of immune response was observed in LPS-treated mice. Therefore, it is implicated that treatment of human with P2Y11 antagonist such as NF157 might be more effective in suppressing an immune response. We suggest that our findings would justify a detailed study of P2Y11 receptor antagonists as therapeutic candidates for treatment of inflammatory diseases including sepsis.

## Supporting Information

Figure S1 **P2Y11 antagonist NF157 blocked ATP-induced increase of cAMP levels in mouse macrophage RAW264.7 cells.** (A) RAW264.7 cells were incubated with 10–100 µM of ATP for 10 min. (B) Cells were pre-incubated with NF157 (50 µM) for 30 min, and then incubated with 100 µM ATP for 10 min. Intracellular cAMP level was measured as described in *Materials and Methods*. Data is expressed as fold increase in cAMP compared with control. Each value represents the mean ± SE (n = 4). A significant difference between the positive control group (ATP) and the indicated group is indicated by *(p<0.05).
(TIF)

Figure S2 **P2Y11 receptor antagonist NF157 suppressed production of IL-6 from peritoneal macrophages.** Peritoneal macrophages were pretreated with NF449 (10 µM), NF157 (50 µM), or KH7 (100 µM) for 30 min. At 24 h after LPS (10 µg/mL) (**A**) or ATP (100 µM) (**B**) stimulation, supernatants were collected and the concentration of IL-6 was measured by ELISA (n = 5). Each value represents the mean ± SE. Significant differences between the positive control group (LPS) and the indicated group are indicated by *(p<0.05) and ***(p<0.001).
(TIF)

Figure S3 **P2X1 antagonist NF449 did not suppressed the increase in serum levels of cytokines in LPS-treated mice.** (**A**–**D**) Male C57BL/6 mice at 6 weeks of age were given LPS (400 µg/head i.p.). NF449 (100 µL of 100 µM) were administered intraperitoneally at 2 h before LPS was injected. Blood samples were collected at 6 h after LPS injection. Serum levels of IL-1ß, IL-6, IL-12 and TNF-alpha were determined as described in *Materials and Methods*. Each value represents the mean ± SE (n = 5).
(TIF)

## References

[1] Cohen J (2002) The immunopathogenesis of sepsis. Nature 420: 885–891. 12490963 10.1038/nature01326

[2] Kilbourn RG, Griffith OW, Gross SS (1993) Pathogenetic mechanisms of septic shock. N Engl J Med 329: 1427–1428. 7692299 10.1056/NEJM199311043291916

[3] Hotchkiss RS, Karl IE (2003) The pathophysiology and treatment of sepsis. N Engl J Med 348: 138–150. 12519925 10.1056/NEJMra021333

[4] Riedemann NC, Guo RF, Ward PA (2003) The enigma of sepsis. J Clin Invest 112: 460–467. 12925683 10.1172/JCI19523 PMC171398

[5] Glauser MP (1996) The inflammatory cytokines. New developments in the pathophysiology and treatment of septic shock. Drugs 52 Suppl 29–17. 10.2165/00003495-199600522-00004 8869831

[6] Angus DC, Linde-Zwirble WT, Lidicker J, Clermont G, Carcillo J, et al (2001) Epidemiology of severe sepsis in the United States: analysis of incidence, outcome, and associated costs of care. Crit Care Med 29: 1303–1310. 11445675 10.1097/00003246-200107000-00002

[7] Martin GS, Mannino DM, Eaton S, Moss M (2003) The epidemiology of sepsis in the United States from 1979 through 2000. N Engl J Med 348: 1546–1554. 12700374 10.1056/NEJMoa022139

[8] Spellberg B, Edwards JE Jr (2001) Type 1/Type 2 immunity in infectious diseases. Clin Infect Dis 32: 76–102. 11118387 10.1086/317537

[9] Nathan CF, Murray HW, Wiebe ME, Rubin BY (1983) Identification of interferon-gamma as the lymphokine that activates human macrophage oxidative metabolism and antimicrobial activity. J Exp Med 158: 670–689. 6411853 10.1084/jem.158.3.670 PMC2187114

[10] Stein M, Keshav S, Harris N, Gordon S (1992) Interleukin 4 potently enhances murine macrophage mannose receptor activity: a marker of alternative immunologic macrophage activation. J Exp Med 176: 287–292. 1613462 10.1084/jem.176.1.287 PMC2119288

[11] Zhang X, Morrison DC (1993) Lipopolysaccharide-induced selective priming effects on tumor necrosis factor alpha and nitric oxide production in mouse peritoneal macrophages. J Exp Med 177: 511–516. 8426119 10.1084/jem.177.2.511 PMC2190891

[12] Gordon S (2003) Alternative activation of macrophages. Nat Rev Immunol 3: 23–35. 12511873 10.1038/nri978

[13] Martinez FO, Helming L, Gordon S (2009) Alternative activation of macrophages: an immunologic functional perspective. Annu Rev Immunol 27: 451–483 PMID: 17972350. 10.1146/annurev.immunol.021908.132532 19105661

[14] Gordon S (2007) The macrophage: past, present and future. Eur J Immunol 37 Suppl 1S9–17. 17972350 10.1002/eji.200737638

[15] Mehta A, Brewington R, Chatterji M, Zoubine M, Kinasewitz GT, et al (2004) Infection-induced modulation of m1 and m2 phenotypes in circulating monocytes: role in immune monitoring and early prognosis of sepsis. Shock 22: 423–430. 15489634 10.1097/01.shk.0000142184.49976.0c

[16] Bozza FA, Salluh JI, Japiassu AM, Soares M, Assis EF, et al (2007) Cytokine profiles as markers of disease severity in sepsis: a multiplex analysis. Crit Care 11: R49. 17448250 10.1186/cc5783 PMC2206478

[17] Lopez-Bojorquez LN, Dehesa AZ, Reyes-Teran G (2004) Molecular mechanisms involved in the pathogenesis of septic shock. Arch Med Res 35: 465–479 PMID: 15631870. 10.1016/j.arcmed.2004.07.006 15631870

[18] Burnstock G (2009) Purinergic signalling: past, present and future. Braz J Med Biol Res 42: 3–8. 18853040 10.1590/s0100-879x2008005000037

[19] Grahames CB, Michel AD, Chessell IP, Humphrey PP (1999) Pharmacological characterization of ATP- and LPS-induced IL-1beta release in human monocytes. Br J Pharmacol 127: 1915–1921. 10482924 10.1038/sj.bjp.0702732 PMC1566177

[20] Piccini A, Carta S, Tassi S, Lasiglie D, Fossati G, et al (2008) ATP is released by monocytes stimulated with pathogen-sensing receptor ligands and induces IL-1beta and IL-18 secretion in an autocrine way. Proc Natl Acad Sci U S A 105: 8067–8072. 18523012 10.1073/pnas.0709684105 PMC2430360

[21] Sutterwala FS, Ogura Y, Szczepanik M, Lara-Tejero M, Lichtenberger GS, et al (2006) Critical role for NALP3/CIAS1/Cryopyrin in innate and adaptive immunity through its regulation of caspase-1. Immunity 24: 317–327. 16546100 10.1016/j.immuni.2006.02.004

[22] Corriden R, Insel PA (2010) Basal release of ATP: an autocrine-paracrine mechanism for cell regulation. Sci Signal 3: re1. 20068232 10.1126/scisignal.3104re1 PMC3085344

[23] Tsukimoto M, Tokunaga A, Harada H, Kojima S (2009) Blockade of murine T cell activation by antagonists of P2Y6 and P2X7 receptors. Biochem Biophys Res Commun 384: 512–518. 19426712 10.1016/j.bbrc.2009.05.011

[24] Sperlagh B, Hasko G, Nemeth Z, Vizi ES (1998) ATP released by LPS increases nitric oxide production in raw 264.7 macrophage cell line via P2Z/P2X7 receptors. Neurochem Int 33: 209–215. 9759915 10.1016/s0197-0186(98)00025-4

[25] Sawada K, Echigo N, Juge N, Miyaji T, Otsuka M, et al (2008) Identification of a vesicular nucleotide transporter. Proc Natl Acad Sci U S A 105: 5683–5686. 18375752 10.1073/pnas.0800141105 PMC2311367

[26] Iwatsuki K, Ichikawa R, Hiasa M, Moriyama Y, Torii K, et al (2009) Identification of the vesicular nucleotide transporter (VNUT) in taste cells. Biochem Biophys Res Commun 388: 1–5. 19619506 10.1016/j.bbrc.2009.07.069

[27] Sathe MN, Woo K, Kresge C, Bugde A, Luby-Phelps K, et al (2011) Regulation of purinergic signaling in biliary epithelial cells by exocytosis of SLC17A9-dependent ATP-enriched vesicles. J Biol Chem 286: 25363–25376. 21613220 10.1074/jbc.M111.232868 PMC3137107

[28] Tokunaga A, Tsukimoto M, Harada H, Moriyama Y, Kojima S (2010) Involvement of SLC17A9-dependent vesicular exocytosis in the mechanism of ATP release during T cell activation. J Biol Chem 285: 17406–17416. 20382737 10.1074/jbc.M110.112417 PMC2878504

[29] Takai E, Tsukimoto M, Harada H, Sawada K, Moriyama Y, et al.. (2012) Autocrine regulation of TGF-beta1-induced cell migration by exocytosis of ATP and activation of P2 receptors in human lung cancer cells. J Cell Sci PMID: 22946048. 10.1242/jcs.104976 22946048

[30] Ben Yebdri F, Kukulski F, Tremblay A, Sevigny J (2009) Concomitant activation of P2Y(2) and P2Y(6) receptors on monocytes is required for TLR1/2-induced neutrophil migration by regulating IL-8 secretion. Eur J Immunol. 39: 2885–2894. 10.1002/eji.200939347 PMC5140286 19735076

[31] Into T, Fujita M, Okusawa T, Hasebe A, Morita M, et al (2002) Synergic effects of mycoplasmal lipopeptides and extracellular ATP on activation of macrophages. Infect Immun. 70: 3586–3591. 10.1128/IAI.70.7.3586-3591.2002 PMC128035 12065499

[32] von Kugelgen I, Harden TK (2011) Molecular pharmacology, physiology, and structure of the P2Y receptors. Adv Pharmacol 61: 373–415. 21586365 10.1016/B978-0-12-385526-8.00012-6

[33] Balogh J, Wihlborg AK, Isackson H, Joshi BV, Jacobson KA, et al (2005) Phospholipase C and cAMP-dependent positive inotropic effects of ATP in mouse cardiomyocytes via P2Y11-like receptors. J Mol Cell Cardiol 39: 223–230. 15893764 10.1016/j.yjmcc.2005.03.007 PMC3471220

[34] Talasila A, Germack R, Dickenson JM (2009) Characterization of P2Y receptor subtypes functionally expressed on neonatal rat cardiac myofibroblasts. Br J Pharmacol 158: 339–353. 19422377 10.1111/j.1476-5381.2009.00172.x PMC2795244

[35] Hara S, Mizukami H, Kuriiwa F, Mukai T (2011) cAMP production mediated through P2Y(11)-like receptors in rat striatum due to severe, but not moderate, carbon monoxide poisoning. Toxicology 288: 49–55. 21777648 10.1016/j.tox.2011.07.001

[36] Schenk U, Westendorf AM, Radaelli E, Casati A, Ferro M, et al (2008) Purinergic control of T cell activation by ATP released through pannexin-1 hemichannels. Sci Signal 1: ra6. 18827222 10.1126/scisignal.1160583

[37] Barbera-Cremades M, Baroja-Mazo A, Gomez AI, Machado F, Di Virgilio F, et al (2012) P2X7 receptor-stimulation causes fever via PGE2 and IL-1beta release. Faseb J 26: 2951–2962. 22490780 10.1096/fj.12-205765

[38] Pelegrin P, Surprenant A (2009) Dynamics of macrophage polarization reveal new mechanism to inhibit IL-1beta release through pyrophosphates. EMBO J. 28: 2114–27. 10.1038/emboj.2009.163 PMC2699392 19536133

[39] Wilkin F, Duhant X, Bruyns C, Suarez-Huerta N, Boeynaems JM, et al (2001) The P2Y11 receptor mediates the ATP-induced maturation of human monocyte-derived dendritic cells. J Immunol 166: 7172–7177. 11390464 10.4049/jimmunol.166.12.7172

[40] Yu J, Sheung N, Soliman EM, Spirli C, Dranoff JA (2009) Transcriptional regulation of IL-6 in bile duct epithelia by extracellular ATP. Am J Physiol Gastrointest Liver Physiol. 296: G563–571. 10.1152/ajpgi.90502.2008 PMC2660176 19136380

[41] Meis S, Hamacher A, Hongwiset D, Marzian C, Wiese M, et al (2010) NF546 [4,4′-(carbonylbis(imino-3,1-phenylene-carbonylimino-3,1-(4-methyl-phenylene)-carbonylimino))-bis(1,3-xylene-alpha,alpha’-diphosphonic acid) tetrasodium salt] is a non-nucleotide P2Y11 agonist and stimulates release of interleukin-8 from human monocyte-derived dendritic cells. J Pharmacol Exp Ther 332: 238–247. 19815812 10.1124/jpet.109.157750

