# Supplementary figures and images for: Autocrine Regulation of Macrophage Activation via Exocytosis of ATP and Activation of P2Y11 Receptor
Source: PLoS One. 2013 Apr 5;8(4):e59778. doi: 10.1371/journal.pone.0059778 (PMC3618444; doi:10.1371/journal.pone.0059778)

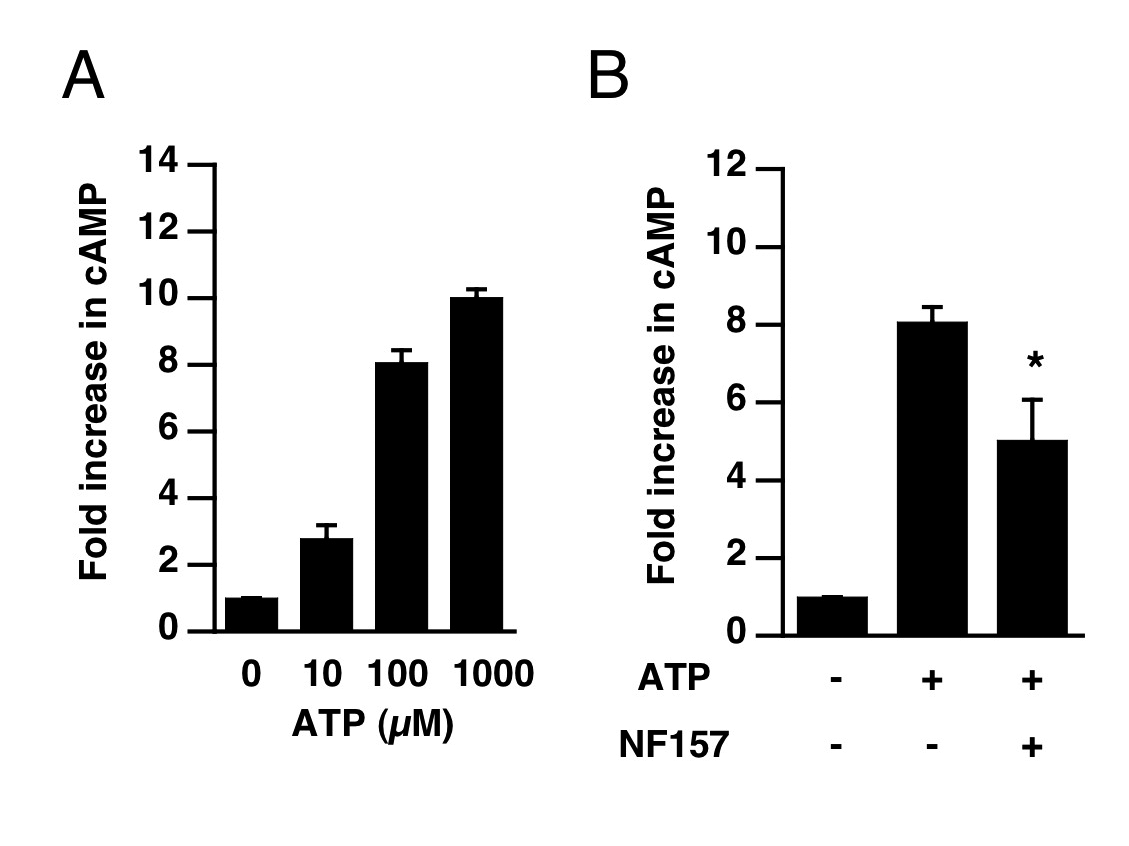

Supplement: Figure S1 — P2Y11 antagonist NF157 blocked ATP-induced increase of cAMP levels in mouse macrophage RAW264.7 cells. (A) RAW264.7 cells were incubated with 10–100 µM of ATP for 10 min. (B) Cells were pre-incubated with NF157 (50 µM) for 30 min, and then incubated with 100 µM ATP for 10 min. Intracellular cAMP level was measured as described in Materials and Methods. Data is expressed as fold increase in cAMP compared with control. Each value represents the mean ± SE (n = 4). A significant difference between the positive control group (ATP) and the indicated group is indicated by *(p<0.05). (TIF) [file pone.0059778.s001.tif]

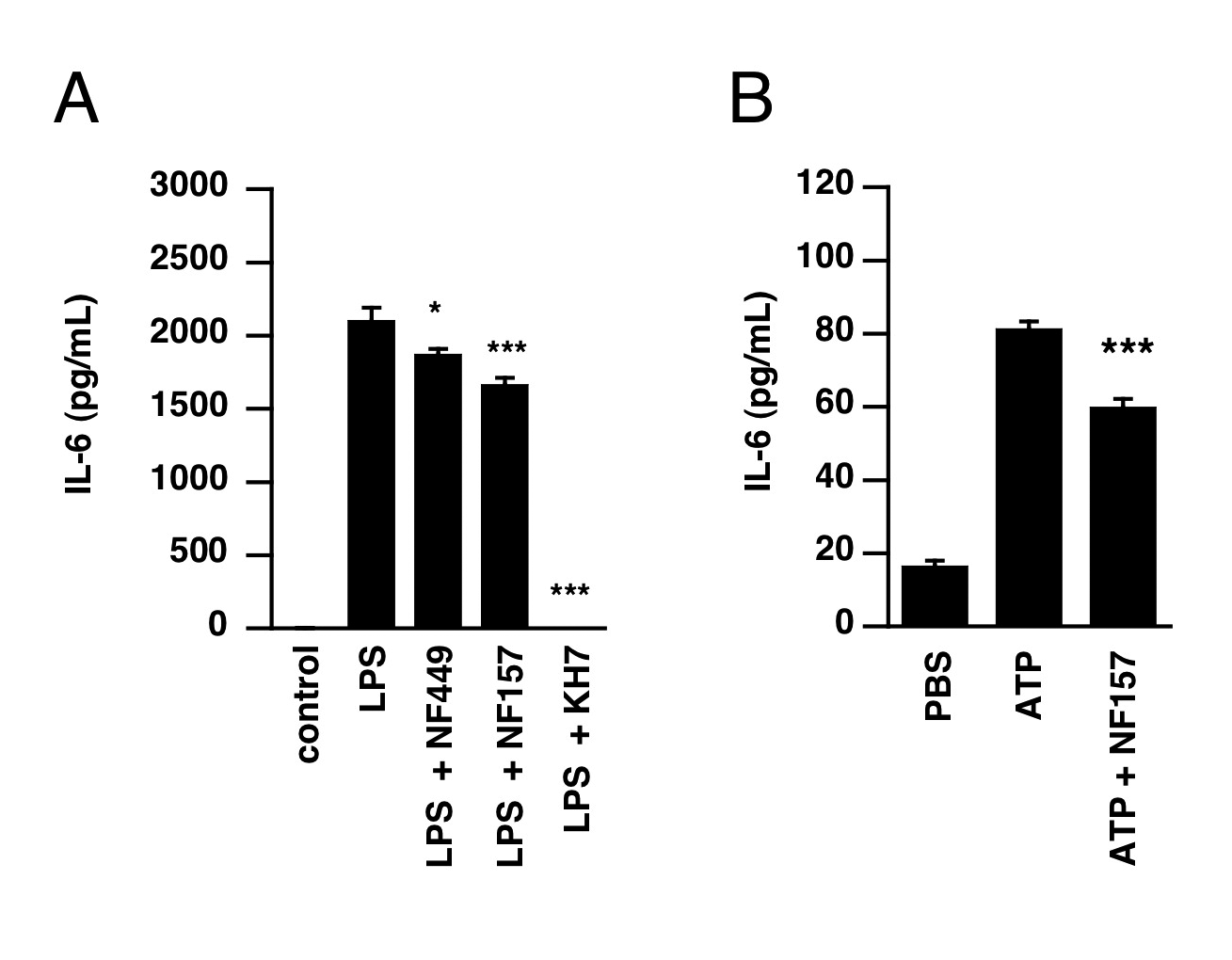

Supplement: Figure S2 — P2Y11 receptor antagonist NF157 suppressed production of IL-6 from peritoneal macrophages. Peritoneal macrophages were pretreated with NF449 (10 µM), NF157 (50 µM), or KH7 (100 µM) for 30 min. At 24 h after LPS (10 µg/mL) (A) or ATP (100 µM) (B) stimulation, supernatants were collected and the concentration of IL-6 was measured by ELISA (n = 5). Each value represents the mean ± SE. Significant differences between the positive control group (LPS) and the indicated group are indicated by *(p<0.05) and ***(p<0.001). (TIF) [file pone.0059778.s002.tif]

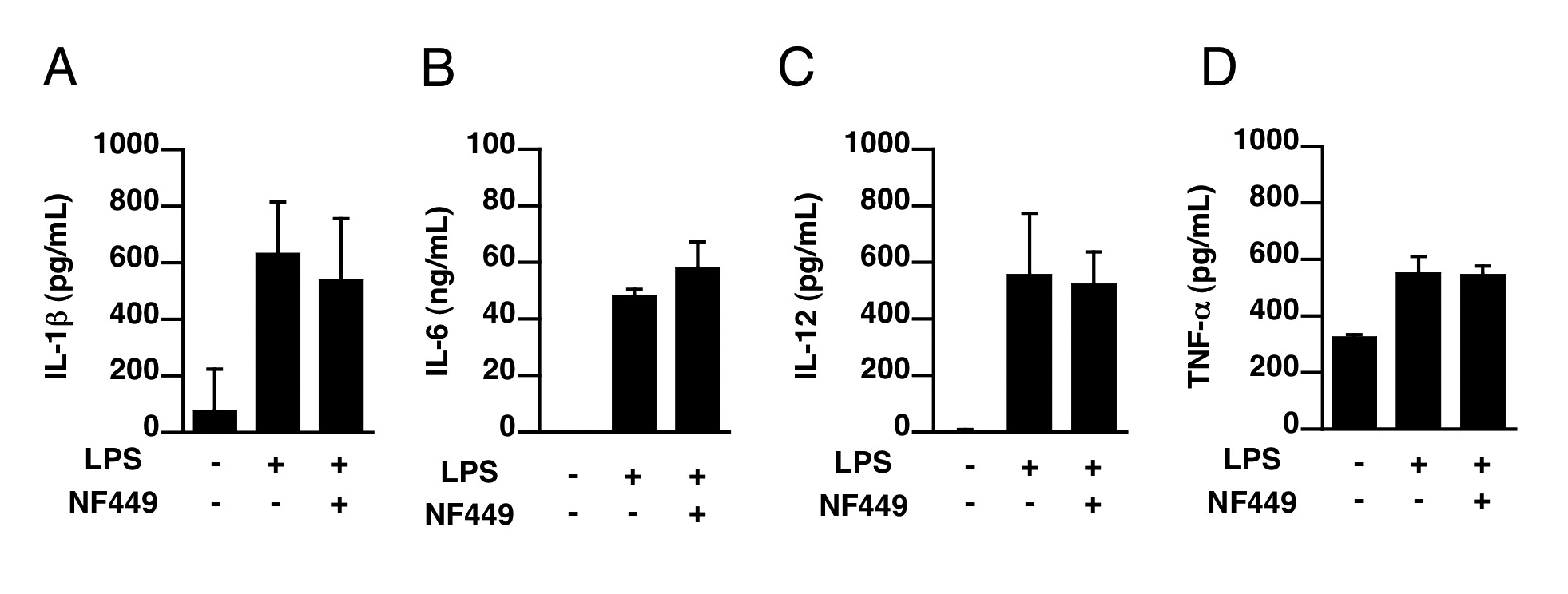

Supplement: Figure S3 — P2X1 antagonist NF449 did not suppressed the increase in serum levels of cytokines in LPS-treated mice. (A–D) Male C57BL/6 mice at 6 weeks of age were given LPS (400 µg/head i.p.). NF449 (100 µL of 100 µM) were administered intraperitoneally at 2 h before LPS was injected. Blood samples were collected at 6 h after LPS injection. Serum levels of IL-1ß, IL-6, IL-12 and TNF-alpha were determined as described in Materials and Methods. Each value represents the mean ± SE (n = 5). (TIF) [file pone.0059778.s003.tif]
